# Tuning the Diradical Character of Pentacene Derivatives via Non-Benzenoid Coupling Motifs

**DOI:** 10.1021/jacs.3c02027

**Published:** 2023-04-26

**Authors:** Tao Wang, Paula Angulo-Portugal, Alejandro Berdonces-Layunta, Andrej Jancarik, André Gourdon, Jan Holec, Manish Kumar, Diego Soler, Pavel Jelinek, David Casanova, Martina Corso, Dimas G. de Oteyza, Jan Patrick Calupitan

**Affiliations:** † Donostia International Physics Center, 20018 San Sebastián, Spain; ‡ Centro de Física de Materiales (CFM-MPC), CSIC-UPV/EHU, 20018 San Sebastián, Spain; § Univ. Bordeaux, CNRS, Centre de Recherche Paul Pascal, CRPP, UMR 5031, 33600 Pessac, France; ∥ CEMES-CNRS, 29 Rue J. Marvig, 31055 Toulouse, France; ⊥ Institute of Physics of the Czech Academy of Sciences, Cukrovarnicka 10, 162 00 Praha, Czech Republic; # Ikerbasque, Basque Foundation for Science, 48009 Bilbao, Spain; ¶ Nanomaterials and Nanotechnology Research Center (CINN), CSIC-UNIOVI-PA, 33940 El Entrego, Spain

## Abstract

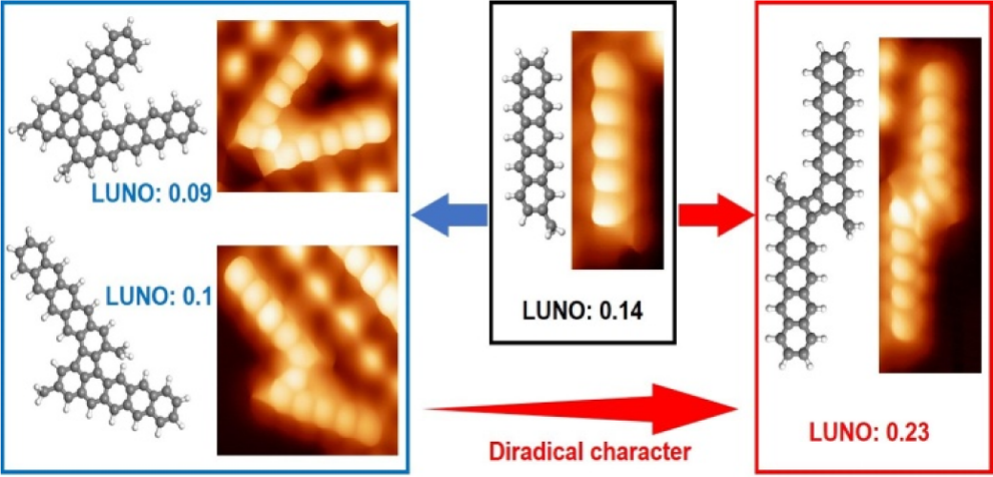

The development of functional organic molecules requires structures of increasing size and complexity, which are typically obtained by the covalent coupling of smaller building blocks. Herein, with the aid of high-resolution scanning tunneling microscopy/spectroscopy and density functional theory, the coupling of a sterically demanded pentacene derivative on Au(111) into fused dimers connected by non-benzenoid rings was studied. The diradical character of the products was tuned according to the coupling section. In particular, the antiaromaticity of cyclobutadiene as the coupling motif and its position within the structure play a decisive role in shifting the natural orbital occupancies toward a stronger diradical electronic character. Understanding these structure–property relations is desirable not only for fundamental reasons but also for designing new complex and functional molecular structures.

## Introduction

The development of substrate-supported organic chemistry under ultra-high vacuum conditions, typically termed “on-surface synthesis”,^1,2^ has allowed in recent years the fabrication and characterization of open-shell polyaromatic hydrocarbons that, to date, were rarely accessible by conventional means.^3,4^ Iconic examples are acenes longer than pentacene,^5,6^ notable for their open-shell diradical character that increases with length,^7−10^ and triangulene, which was hypothesized as a triplet-ground-state molecule as early as the 1950s,^11^ but it was not synthesized in its unprotected form until 2017.^12^

**Scheme 1:**
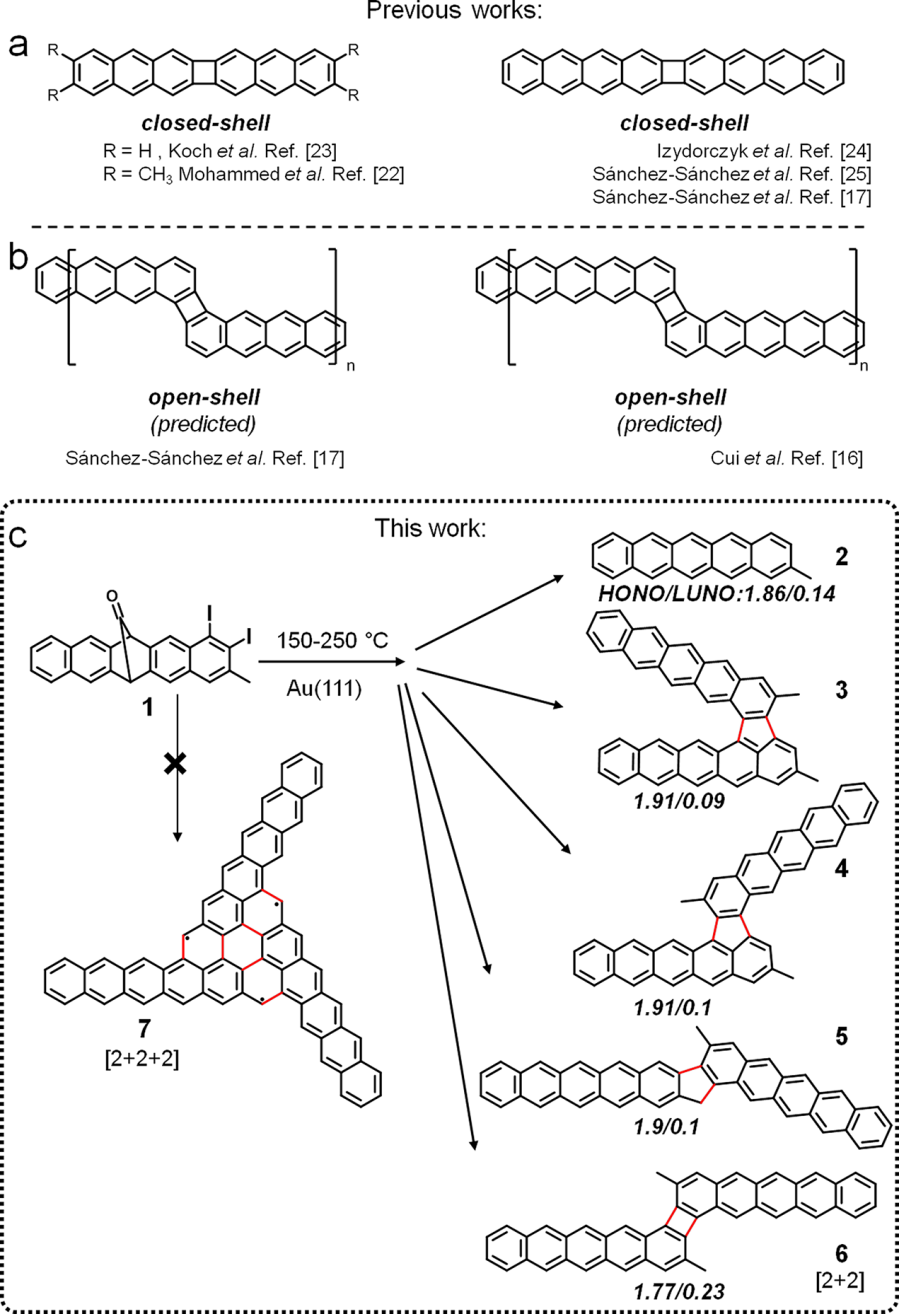
Previous Works on Acenes Fused by a Non-Benzenoid Four-Membered Ring in a (a) Linear and (b) Staggered Arrangement; (c) Molecular Structure of the Reactant and of Possible End Products upon Surface-Supported Reactions New intermolecular bonds are marked in red. Natural orbital occupancies of compounds **2–6** indicated below.

In a continuous search for increasingly complex structures, such molecules have been further combined into oligomers or polymers,^13−24^ evidencing an impressively rich phenomenology and varying properties with a strong dependence on the coupling motifs. Scheme 1a illustrates anthracenes^22,23^ and tetracenes^17,24,25^ coupled at C2 and C3 positions, forming dimers fused through a non-benzenoid cyclobutadiene ring in a straight linear motif (see Scheme S1a for numbering guide on acene). Such acenes bridged by a four-membered ring in a linear fashion were found to have a closed-shell electronic structure.^17,22−25^ Meanwhile, tetracene^16,17^ and pentacene^16^ units coupled on C1 and C2 positions, thus bridged by cyclobutadiene in an angular direction, were predicted to have an open-shell character (Scheme 1b). Likewise, polyaromatic hydrocarbons having five-membered rings were found to display closed or open-shell characters according to how aromatic units are arranged around the non-benzenoid ring.^26,27^ Tuning functionalities for spintronic applications, therefore, require an understanding of these structure–property relations, concomitant with the fabrication of various acene derivatives with different non-benzenoid coupling motifs.

Doubly halogenated arenes are especially attractive building blocks since their in situ dehalogenation results in rich on-surface chemistries,^28,29^ ideal for exploring a diverse set of larger structures and tuning their properties. Herein, we have synthesized the ortho-dihalogenated pentacene derivative (6R)-1,2-diiodo-3-methyl-6,13-dihydro-6,13-methanopentacen-15-one (**1**, Scheme 1c) as a precursor to fabricate pentacene dimers fused through different coupling motifs on Au(111) and study their effect in the resulting electronic structure. Although decarbonylation and dehalogenation may simply lead to **2** (Scheme 1), coupling could result in dimer products with various addition motifs. Since **1** has two halogenated sites, various monocoupling reactions followed by cyclodehydrogenations^30,31^ could result in fused dimer products **3**–**5** (Scheme S1c). In addition, since the activated carbons are in ortho-positions, [2 + 2] dimerization could lead to end product **6**.^17,22−25^ In turn, a potential [2 + 2 + 2] cyclotrimerization^17,22−25^ would lead to end product **7**.

Compounds **3**, **4**, **5**, and **6** could be seen as dimeric pentacene units connected by non-benzenoid motifs: **3**, **4**, and **5** have five-membered rings, while **6** has a four-membered ring. Meanwhile, product **7** can be rationalized as a triangulene core with additional acene arms. Among these, **3** was found to be the major product on the Au(111) surface, **6** was found to be the minor kinetic product, and **7** was not obtained. We rationalize this outcome from thermodynamic and kinetic arguments. Further, by combining scanning tunneling microscopy and spectroscopy (STM/STS) with theoretical calculations, we show that **3**, **4**, and **5** all have similar occupied–unoccupied orbital gaps and natural orbital occupation numbers (a measure for the radical character of molecules^32,33^). Instead, **6** shows a dramatically reduced gap, along with a reduced (increased) occupation number of its highest occupied (lowest unoccupied) natural orbital that denotes an increased diradical character. We rationalize this by molecular orbital analyses and (anti-)aromaticity arguments, offering molecular design principles for fine-tuning the diradical character of molecular materials.

## Results

### On-Surface Synthesis

**Figure 1:**
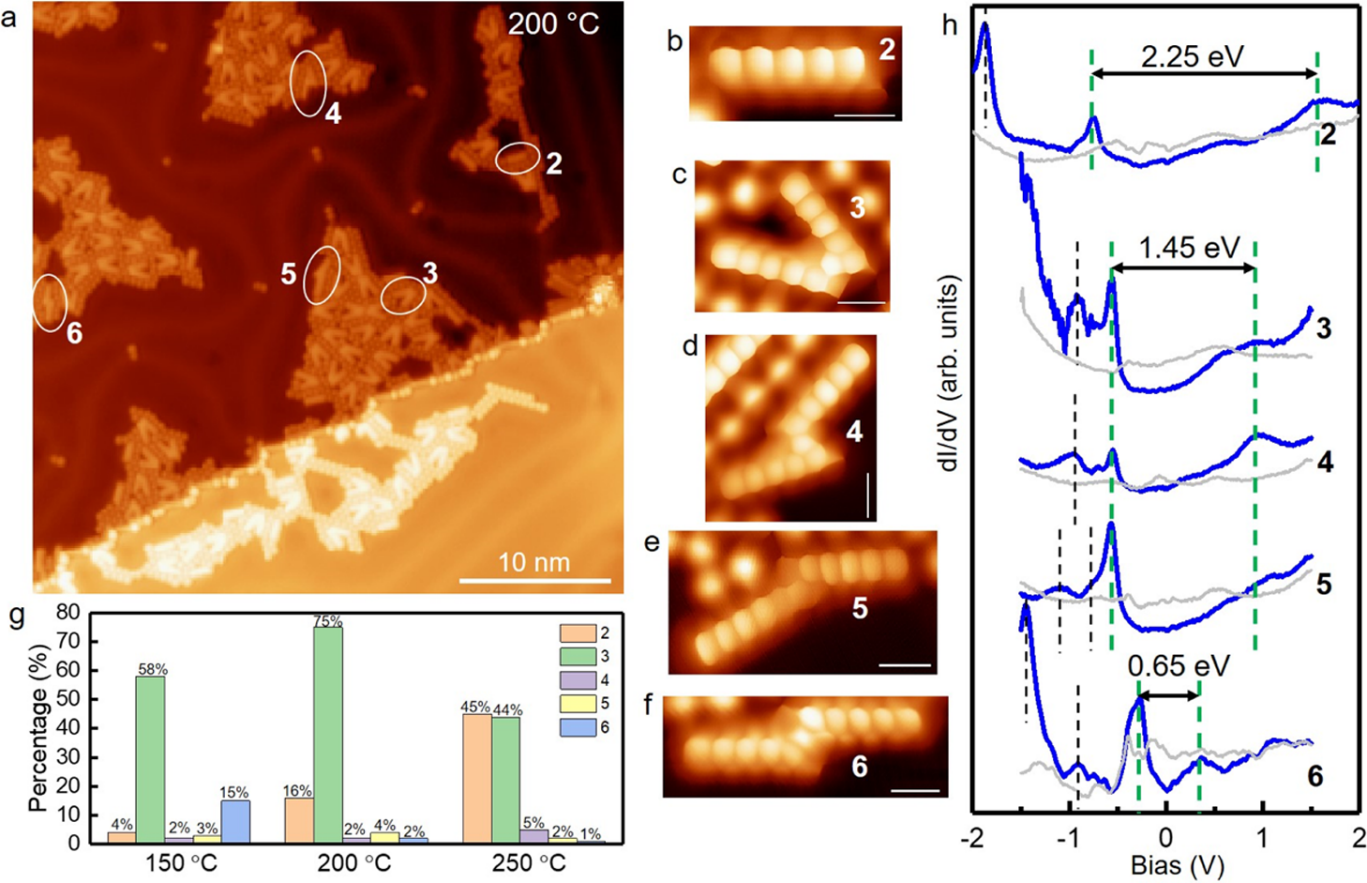
(a) Overview STM image (*U* = 500 mV; *I* = 100 pA) of a sample prepared by depositing precursor molecule **1** on the Au(111) surface held at 200 °C. Bond-resolving STM images (*U* = 5 mV at constant height mode) of (b) **2**, (c) **3**, (d) **4**, (e) **5**, and (f) **6**. (g) Histogram of the product distribution at the various temperatures tested, obtained from more than 300 molecules at each temperature. Missing percentages correspond to unassigned structures. (h) Representative d*I*/d*V* spectra of structures **2**–**6**, with HOMO and LUMO positions marked by dashed green lines and positions of other orbitals by black dashed lines as a guide to the eye. Scale bars in (b–f) are 5 Å.

On-surface synthesis commonly proceeds by depositing a reactant precursor onto clean metallic substrates held at room temperature (RT), followed by annealing to trigger surface-supported reactions.^1,2^ Evaporating **1** onto the Au(111) substrate at RT led to an iodine-decorated surface without molecular products (Figure S1), which hinted at an initial dehalogenation followed by molecular desorption. Desorption of such dehalogenated pentacene derivatives at relatively low temperatures indicates weak molecule–substrate interactions due to the decoupling effect associated with the non-planarity introduced by the sp^3^ carbon atoms at the carbonyl and methyl groups.^34^ However, deposition of **1** onto Au(111) held at higher temperatures (≥150 °C) promoted molecular coupling before desorption and thus resulted in surfaces decorated with molecular structures, along with associated iodine adatoms which aggregate the products into close-packed islands by hydrogen bonding.^35,36^Figure 1a shows a large-scale STM image of Au(111) held at 200 °C during precursor deposition (see Figure S2 for a better recognition of the various molecular species present). The absence of **1** indicates that dehalogenation and decarbonylation readily occur at or below the probed temperatures,^37,38^ resulting in intermolecular coupling products (see Figure S3 for deposition on Au(111) held at 150 and 250 °C).

A limited number of molecular products was observed. Figure 1b–f shows bond-resolving STM images obtained with CO-functionalized probes that allow assigning the various products to structures **2**–**6** (Scheme 1). Whereas decarbonylation merely caused sp^3^ to sp^2^ rehybridization of central carbon atoms (C6 and C13), dehalogenation generates two neighboring radicals that drive intermolecular couplings, forming products **3**–**6**. At all temperatures probed, **3** was found to dominate the yield of the dimers, but the amount of **6** increased at 150 °C (Figure 1g). Since lower temperatures stabilize kinetically preferred products,^39,40^**6** must be the kinetic product—activated aryl carbons readily form covalent bonds on Au(111) at 150 °C^41−43^ (i.e., with low energy barriers). Yet, **6** remained a minor product, probably because it requires two pairs of suitable carbon sites on two pentacene units to be aligned for appropriate bond formation. This can be extended to explain why the [2 + 2 + 2] cyclotrimerization into **7** would not proceed—three pentacene units have even less chances to arrange toward pairwise tri-coupling on a hot reactive substrate, which is additionally sterically hindered by methyl groups (repulsion between methyl and aryl hydrogens in the other pentacene unit). On the other hand, **3** has less geometric constraints, requiring only one pair of carbon sites to couple before subsequent dehydrogenation (Scheme S1b). We note that **3** might also form from dehalogenated pentacene derivatives by other pathways that involve H-migration^44−46^ to the adjacent phenyl rings (Figure S4), allowing more possibilities for its formation. For comparison, the formation of **4**, a trans-isomer of **3**, involves larger steric hindrance (repulsion between methyl and aryl hydrogens) and thus only appears as a minor product. DFT calculations revealed that **3** is more stable than **6** by 2 eV (Figure S5), in line with **3** being the major thermodynamic product.

Comparison of their electronic energy gaps points in the same direction. Figure 1h shows representative conductance spectra of products **2**–**6**, with the highest occupied molecular orbital (HOMO) and lowest unoccupied molecular orbital (LUMO) marked by green dashed lines as guides to the eye. It should be remarked that although the spectra are obtained on molecules surrounded by iodine atoms, we have previously shown by systematically comparing molecules with and without surrounding halogens that their presence has no effect on the HOMO–LUMO gap values nor on the density of states distributions of molecular orbitals and only causes a minor (rigid) energy shift of occupied and unoccupied states.^47,48^ As π-conjugation extends, the gap shrinks: the largest was found for monomer **2** (2.25 eV) but decreased to 1.45 eV for the fused dimer derivatives **3**, **4**, and **5**. Intriguingly, **6** displayed a much smaller gap of 0.65 eV, which supports that **6** is kinetically but not thermodynamically preferred—larger gaps are associated with more stable structures.

The HOMO–LUMO gap reduction to less than half, from **3**–**5** to **6**, is surprising since **6** has the same extent of π-conjugation as the other fused products. In the following, we analyze in greater detail the electronic properties of **3**, being the most common dimer structure, and compare them with those of **6**.

### Electronic Structure and Non-Benzenoid Rings

**Figure 2:**
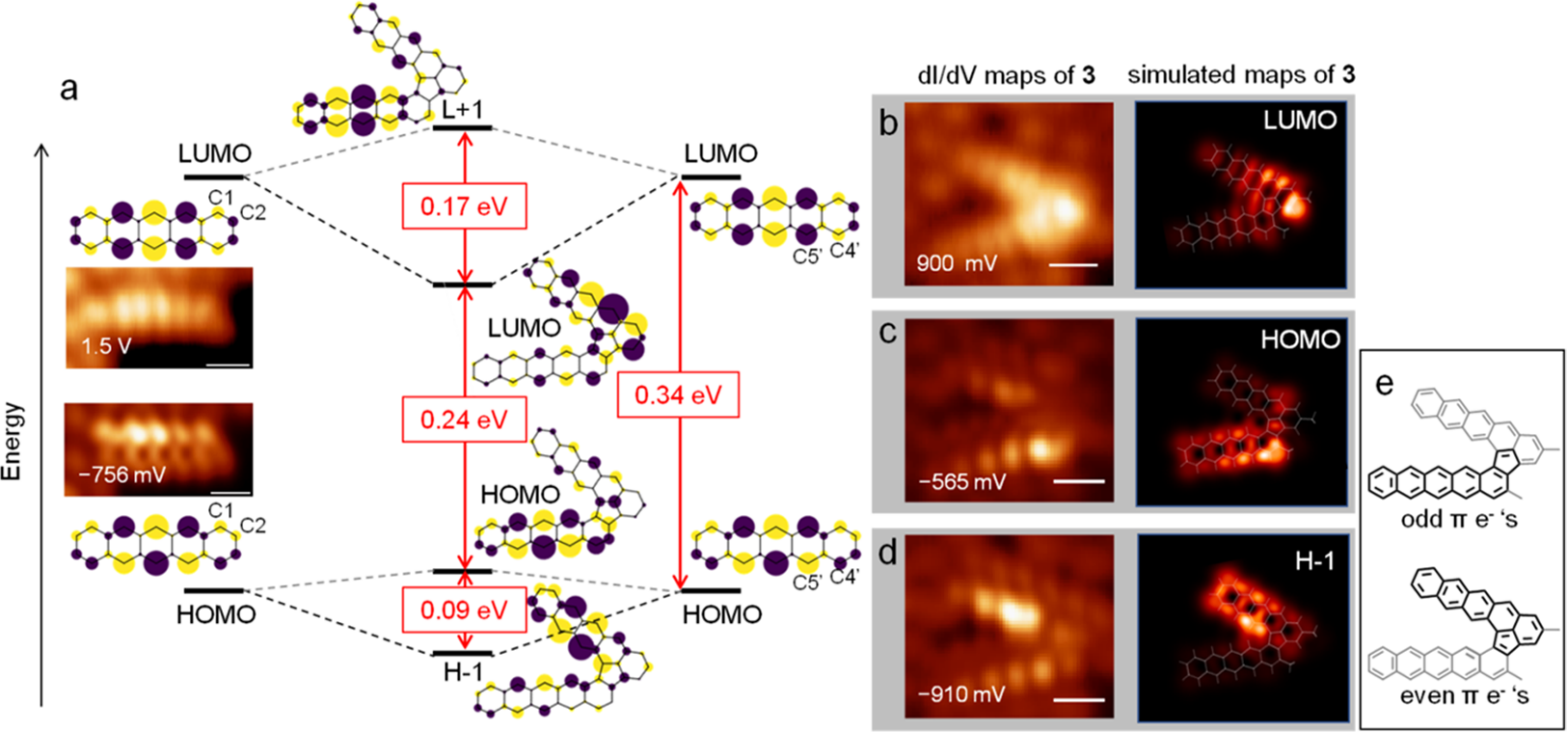
(a) Hückel model molecular orbitals of the monomer and of dimer **3**, with the horizontal lines marking their relative energies to scale. Conductance maps of the monomer **2**’s HOMO and LUMO are shown as insets. Relevant carbons that serve as linking atoms are marked. Experimental (left) and simulated (right) conductance maps of dimer **3** are shown in (b) for the LUMO, (c) for the HOMO, and (d) for the HOMO–1 (indicated as H-1 in the figure). (e) Schematic partition of the dimer structure assigning the linking ring to one or another monomer. All the scale bars in STM images are 5 Å.

Figure 2a displays the experimentally measured d*I*/d*V* maps of **2** and **3** along with simulated Hückel model orbitals. Such a model only considers π-electrons and thus obviates methyl functionalization. The fact that the energy gap and the measured conductance maps corresponding to the HOMO and LUMO of **2** (Figure 2a) perfectly fit those of pentacene underlines the negligible influence of the methyl group on the frontier orbitals and justifies this approximation.^49^ The measured reduction in HOMO–LUMO gap from **2** to **3** by a third (2.25 eV for **2**, 1.45 eV for **3**, Figure 1h) is also qualitatively reproduced by this model. Nevertheless, to provide further support, we have also used DFT calculations, which render similar frontier orbital topologies and orbital energy gaps for molecules with and without methyl functionalization (see Figures S6–S8). The simulated d*I*/d*V* images^50^ associated with their low energy molecular orbitals (LUMO, HOMO and HOMO–1) are in very good agreement with the conductance maps measured for **3** at 900 mV (Figure 2b), −565 mV (Figure 2c), and −910 mV (Figure 2d), respectively.

To visualize the molecular orbital diagram of dimer **3** as arising from linear combinations of the frontier orbitals of monomer **2**, we stick to the simple Hückel model. As illustrated in Figure 2a, the bonding (dark dashed lines) and anti-bonding (gray dashed lines) combinations of **2**’s HOMOs result in the HOMO-1 and HOMO of **3**, while the bonding and anti-bonding combinations of the **2**’s LUMOs result in the LUMO and LUMO+1 of **3**. The strength of orbital overlap between those of the monomers dictate the magnitude of stabilization (or destabilization) of their bonding (or anti-bonding) combinations. Thus, this determines the extent of HOMO–LUMO gap reduction upon fusion as it affects (1) the destabilization of the dimer’s HOMO with respect to the monomer’s HOMO and (2) the stabilization of the dimer’s LUMO with respect to the monomer’s LUMO. If we focus on the monomer’s LUMO, the wavefunctions at the linking atoms display opposite signs on both monomers (i.e., C1 and C2 have opposite signs, C4′ and C5′ have opposite signs), resulting in favorable orbital overlap so that the bonding (LUMO of **3**) and anti-bonding (LUMO+1 of **3**) combinations have a relatively large energy difference (0.17 eV). This changes for the bonding (HOMO–1 of **3**) and anti-bonding (HOMO of **3**) combinations of the monomer’s HOMO as the linking atoms on one monomer display the same sign and opposite signs on the other monomer (i.e., C1 and C2 have the same signs but C4′ and C5′ have opposite signs). This automatically prevents straightforward bonding and antibonding configurations and requires instead combinations with a quasi-null wavefunction amplitude on some of the linking atoms, resulting in a weaker orbital overlap and thus smaller energy difference (0.09 eV) between HOMO – 1 and HOMO. Therefore, the reduction of the HOMO–LUMO gap upon fusion to form **3** could be attributed more to the stabilization of the LUMO than the destabilization of the HOMO with respect to those of the monomer.

The relatively weak orbital interactions could also be understood from the symmetry mismatch of the monomer’s orbitals and the dimer structure. This prevents electron delocalization over the whole structure and causes bonding and antibonding combinations to be primarily located on one monomer. The monomer side onto which those states localize could be rationalized by considering that combining a pentacene unit with a non-benzenoid five-membered ring cycle may result in either an odd or even number of π electrons (Figure 2e). Molecular orbitals which localize on the pentacene unit with the even-numbered π electron configuration are lower in energy than the odd-numbered π electron configuration. Although orbitals also extend partly on the other monomer such that the π conjugation is maximized, a distribution that presents an odd number of electrons is expected to display a higher energy due to an extra electron that cannot bind pairwise.^4^ To illustrate this, the HOMO–1 of **3** is mostly located on the pentacene moiety that is combined with only two more carbons (equivalent to two additional π-electrons) to form the five-membered ring as opposed to the HOMO, which is localized on the other pentacene unit that needs three carbon atoms to form the five-membered ring. This is also valid for the unoccupied orbitals, so the LUMO is localized on the same side as the HOMO–1 and the LUMO+1 as the HOMO. These trends and analyses are equally valid for other dimer products that contain five-membered ring motifs (**4** and **5**; Figures S9 and S10). The chemical structure of the latter is confirmed with tip-induced dehydrogenation experiments shown in Figure S11.

**Figure 3:**
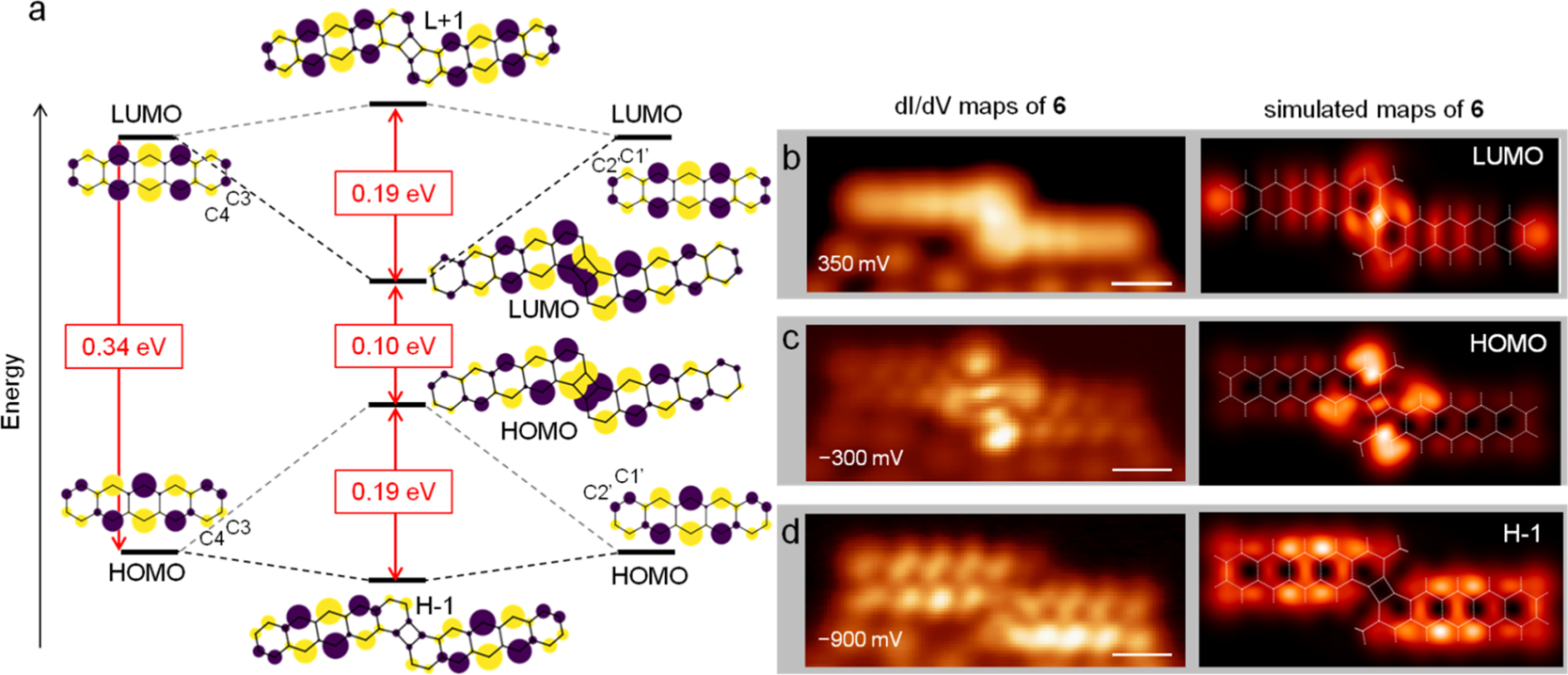
Hückel model molecular orbitals of the monomer and of dimer **6**, with the horizontal lines marking their relative energies to scale. Experimental (left) and simulated (right) conductance maps of **6** are shown in (b) for the LUMO, (c) for the HOMO, and (d) for the HOMO–1 (indicated as H-1 in the figure). All the scale bars are 5 Å.

The scenario changes dramatically for product **6**, which couples the two monomers via carbon atoms that result in bonding and antibonding combinations symmetrically distributed over the whole molecule (Figure 3a). Using the same analysis as above, we note how the symmetry of one-electron states at the linking atoms on monomer **2** is the same for both the HOMO and LUMO. That is, for the HOMO of **2**, both pairs C3/C4 and C1′/C2′ have the same signs, while for the LUMO, both pairs C3/C4 and C1′/C2′ have opposite signs. It results in a large energy difference between bonding and anti-bonding combinations of both HOMO and LUMO (so that for **6**, both LUMO+1/LUMO and HOMO/HOMO–1 have a 0.19 eV energy difference).

Consequently, the HOMO–LUMO gap of **6** shrinks to about one-third of that of the monomer (Figure 3a), which is again in qualitative agreement with experiments (Figure 1h). Importantly, conductance maps obtained at the observed resonance energies (Figure 3b–d) agree with the simulated images of HOMO–1, HOMO, and LUMO, including the particularly strong intensity around the four-membered ring for the latter two.

## Discussion

Experimental fingerprints of open-shell character, like Kondo resonances or spin-flip excitations, have been measured on many organic molecules.^3,4^ However, on higher acenes, this has not been the case in spite of notable efforts,^5−10^ and the discussion on their open- or closed-shell character has been focused on theoretical calculations and their HOMO–LUMO gap since low values of the latter are known to promote the presence of unpaired electrons in molecular materials.^4,27^ The measured 0.65 eV gap of product **6** can indeed be considered very low, almost half that of decacene^9^ or undecacene^10^ that have similar extents of π-conjugation (±2 π-electrons, 10–11 rings) and are considered as open-shell molecules.

Although an open-shell character had been readily predicted for acene dimers^16^ and polymers^16,17^ with linking motifs like those of **6**, its strikingly small HOMO–LUMO gap had not yet been experimentally accessed to date. However, at this point, we would like to point out that the open- or closed-shell nature of molecules is a property allowing for a gradual transition from one to another over a continuous range.^32,33,51−54^ The open-shell character of molecules can be quantified by the natural orbital (NO) occupation numbers, with fractional occupancies indicative of the open-shell nature.^32^ Purely closed-shell (no unpaired electrons) systems exhibit NOs with occupancies of 2 (occupied) and 0 (unoccupied), while half-occupied frontier NOs (HONOs and LUNOs) are indicative of pure radical (one unpaired electron) or polyradical (multiple unpaired electrons) molecules.^32,33,51^ We have thus performed additional calculations to access these occupancies for molecules **2**–**6**. It should also be noted that different calculations may lead to disparate results, and we opt for presenting in the following the values obtained from quantum chemistry complete active space CAS(8,8) calculations, which has been considered to be more reliable but provide notably lower open-shell character than DFT(PBE0) calculations (see Table S1).

As expected, monomer **2** shows comparable HONO/LUNO occupancies (1.86/0.14) to those of pentacene without the methyl functionalization (1.84/0.16). However, it is surprising to see that the occupancy numbers shift toward a pure closed-shell character in dimers **3** (1.91/0.09), **4** (1.91/0.1), and **5** (1.9/0.1), reaching values comparable to those of naphthalene or anthracene (see Table S1). That is, the linkage via five-membered rings reduces the diradical character of the acene dimers.

**Figure 4:**
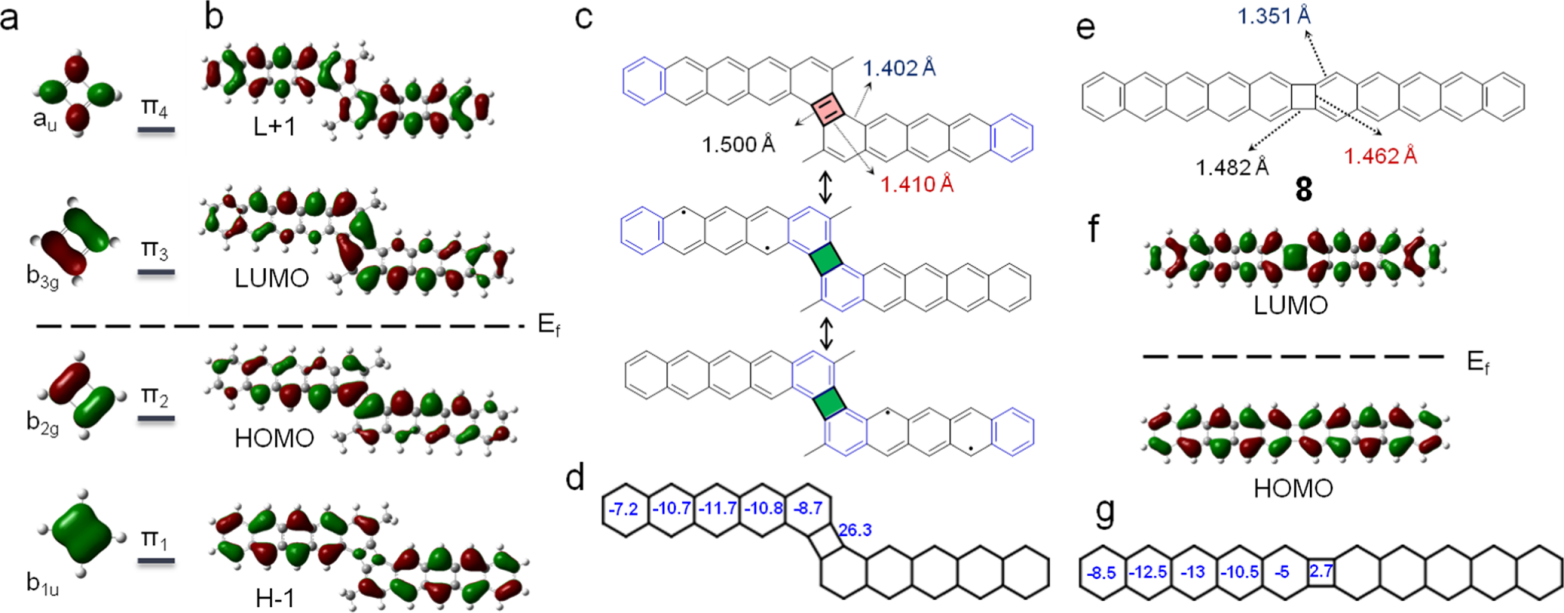
(a) DFT-calculated molecular orbitals of cyclobutadiene. (b) Spin unrestricted calculations of spin-α HOMO–1, HOMO, LUMO and LUMO+1 of **6**. The dashed line marks the Fermi level and the associated state occupancy. (c) Resonance forms of **6** in a closed-shell configuration (top) and open-shell configuration (bottom), showing the calculated length of some relevant bonds. The four-membered ring shaded in red has a cyclobutadiene character, while the bottom resonance structures with the four-membered ring in green have a radialene structure. Clar sextets appear in blue. (d) NICS(1) values calculated on each of the rings of structure **6**. (e) Resonance form of a pentacene dimer linked with a four-membered ring in a colinear manner (structure **8**), along with the calculated length of some relevant bonds. (f) Calculated frontier orbitals of **8**. (g) NICS(1) values on each of the rings of structure **8**.

The opposite happens with dimer **6**, for which the HONO/LUNO occupancy shifts in the open-shell direction to 1.73/0.27, increasing the radical character to values comparable to those of heptacene (see Table S1). In the following, we relate this shift with the particular four-membered ring linking motif. The four-membered ring made by sp^2^ carbon atoms, called cyclobutadiene,^55^ is a well-studied molecular system. It is the archetypical antiaromatic molecule with 4n electrons lacking aromatic stabilization. Whereas its 4-fold symmetric form is a diradical with singly occupied π_2_ and π_3_ states, in its Jahn–Teller distorted stabilized form, the π_2_ and π_3_ states become doubly occupied and fully empty, respectively (Figure 4a).^56^

If we now pay attention to the orbitals of **6** (Figure 4b), particularly at the π-orbital contributions at the four-membered ring, we can recognize the pattern of cyclobutadiene orbitals in the same order and filling, which hints at its destabilizing contribution in **6**. Calculations reveal the bonds that link the two pentacene moieties to be very long (1.5 Å) and thus clearly of single bond character, while the bonds on the phenyl rings are much shorter (1.41 Å) with a double bond-character, akin to cyclobutadiene (Figure 4c). The double bonds on the four-membered ring join four p_*z*_ electrons and therefore render it with an antiaromatic character (top resonance structure, Figure 4c). Confirmation thereof is obtained from NICS(1) calculations, which reveal a large positive (antiaromatic) value on the four-membered ring (Figure 4d; see Table S2 for details). Meanwhile, the neighboring zig-zag bonds (1.402 and 1.410 Å) are comparable to the bond length (1.39 Å) of benzene, which hints at contributing resonant forms that display interchangeable double and single bonds. Rearranging the π-bonds results in a resonant structure that removes the π-electrons from the four-membered ring, avoiding its destabilizing contribution,^57−60^ while at the same time facilitating the generation of diradicals on one or another pentacene moiety and concomitant with the generation of an additional Clar sextet (middle and bottom resonance structures in Figure 4c).

It is instructive to compare **6** with a pentacene dimer that is equally linked by a four-membered ring, but in a collinear arrangement (**8**, Figure 4e). In this case, calculations predict the structure to have a negligible radical character despite its comparable coupling motif, which is also in line with the experiments on similar linearly fused acene dimers and polymers.^17,24^ This can be understood again from its resonance structures, which in a closed-shell form (ground state) require no π-bonds on the four-membered ring and thus no destabilizing antiaromatic effects. Indeed, calculations reveal all bonds of the four-membered ring to be relatively long (Figure 4e) and thus of a single-bond character, while the neighboring bond on the phenyl ring is much shorter (1.351 Å) and confirms its fixed double bond character, as in a radialene moiety.^57^ The same conclusions could be drawn from the calculated molecular orbitals. Focusing again on the four-membered ring of the linear dimer, the local distribution patterns of the frontier π-orbitals do not resemble those of cyclobutadiene (Figure 4f) and can therefore no longer be associated with the cyclobutadiene’s destabilizing effect. The same may be concluded from the close to zero NICS(1) value on that ring (Figure 4g). Such NICS(1) value reflects the single-bond character of the connection between the acene units^20^ and consequently (with the associated phenyl rings) the radialene character^52^ that causes the conjugation between the two pentacene monomers to be weak.^24^

## Conclusions

In conclusion, we have studied the surface-supported formation of sterically demanded pentacene-dimers made out of pentacene derivatives. We observe how the dimer structure linked by a four-membered ring (**6**) is only stabilized kinetically and remains a minor product, whereas a thermodynamic driving force favors other products linked via five-membered rings (mainly **3**). A detailed analysis of their respective electronic properties reveals dimers **3**–**5** to have a reduced diradical character as compared to monomer **2**. In contrast, it increases for dimer **6**, which is attributed to the destabilizing contribution of the cyclobutadiene coupling motif. The same coupling motif at a different position in dimer **8**, however, does not have the same destabilizing effect, which is understood from a qualitative analysis of the structural and electronic properties right at the four-membered ring. This study offers insights into how antiaromaticity could promote the diradical character of hydrocarbons, thus providing design principles not only for spintronics applications but also, more generally, for organic photovoltaics, optoelectronic materials, and chemical reactivity, in which organic diradicals are finding increasing relevance.^32,33,51−54^

## Supporting Information Available

Experimental and theoretical methods, additional STM images, and calculated results (PDF)
