# Supplementary material for: Tuning the Diradical Character of Pentacene Derivatives via Non-Benzenoid Coupling Motifs
Source: J Am Chem Soc. 2023 Apr 26;145(18):10333–41. doi: 10.1021/jacs.3c02027 (PMC10176464; doi:10.1021/jacs.3c02027)
Supplement: Supplementary file 1 — ja3c02027_si_001.pdf [file ja3c02027_si_001.pdf]

Supplementary Information

Tuning the Diradical Character of Pentacene Derivatives *via* non-Benzenoid Coupling Motifs

Tao Wang,[1,2,†] Paula Angulo-Portugal,[2,†] Alejandro Berdonces-Layunta,[1,2] Andrej Jancarik,[3,*] André Gourdon,[4] Jan Holec,[4] Manish Kumar,[5] Diego Soler,[5] Pavel Jelinek,[5] David Casanova,[1,6] Martina Corso,[1,2] Dimas G. de Oteyza,[1,7,*] and Jan Patrick Calupitan[1,2*‡]

[1] Donostia International Physics Center, 20018 San Sebastián, Spain

[2] Centro de Física de Materiales (CFM-MPC), CSIC-UPV/EHU, 20018 San Sebastián, Spain

[3] Univ. Bordeaux, CNRS, Centre de Recherche Paul Pascal, CRPP, UMR 5031, 33600 Pessac, France

[4] CEMES-CNRS, 29 Rue J. Marvig, 31055 Toulouse, France

[5] Institute of Physics of the Czech Academy of Sciences, Cukrovarnicka 10, 162 00, Czech Republic

[6] Ikerbasque, Basque Foundation for Science, 48009 Bilbao, Spain

[7] Nanomaterials and Nanotechnology Research Center (CINN), CSIC-UNIOVI-PA, 33940 El Entrego, Spain

*correspondence to: andrej.jancarik@crpp.cnrs.fr, d.g.oteyza@cinn.es, jan.calupitan@sorbonne-universite.fr

‡ Present Address: Sorbonne Université, CNRS, Institut Parisien de Chimie Moléculaire, F-75005 Paris, France

STM measurements

STM measurements were performed using a commercial Scienta-Omicron LT-STM at 4.3 K. The system consists of a preparation chamber with a typical pressure in the low 10^{-10} mbar regime and a STM chamber with a pressure in the 10^{-11} mbar range. The Au(111) crystal was cleaned *via* two cycles of Ar^+ sputtering and annealing at 720 K. The precursor molecule **1** was evaporated from a home-built molecular evaporator at 473 K. The efficient molecular flux is approximately 0.1 monolayer per minute on the Au(111) surface.

All STM and STS measurements were performed at 4.3 K. To obtain BR-STM images, the tip was functionalized with a CO molecule that was picked up from the Au(111) surface. CO was dosed into the STM chamber *via* a leak valve at a pressure of approximately 1×10^{-8} mbar. For adsorption onto the sample, the STM thermal shields were opened and closed again when reaching a sample temperature of 7.0 K. Three such shield opening cycles were typically applied. CO can be picked up with a metallic tip by scanning with a high current and negative bias (*e.g.* I = 1 nA, U = −0.5 V). dI/dV measurements were recorded with the internal lock-in of the Nanonis electronics. The oscillation frequency used in experiments is 731 Hz and the amplitude is 20 mV for all STS and dI/dV maps shown in the manuscript.

Calculations

The Hückel orbital energies were obtained using the orbital parameters α (the energy of an electron in a p-orbital) and β (the interaction energy between two orbitals) from the reported literature.[1] Representation of Hückel orbitals was done by plotting 2D spherical atomic orbitals proportional to the atomic coefficient in each molecular orbital.

DFT calculations on molecule **6** were performed using the Gaussian 16[2] suite of programs and visualized by GaussView. The basis set 6-311+G(d,p) was used. The closed-shell configurations were first optimized using M06-2X and B3LYP functionals and then the stability of the closed-shell wavefunction was checked by using the *stable=opt* keyword at the same level of theory. For **6**, M06-2X revealed a stable closed-shell structure while B3LYP yielded a diradical anti-ferromagnetic structure which is 77 meV (with a relatively high spin contamination $\langle S^2 \rangle = 1.09$) more stable than the closed-shell configuration. This hints at the diradical character of **6**. Meanwhile, compounds **2-5** were found to have stable closed-shell wavefunctions using both functional. NICS values were calculated at the same level of theory.

Multi-reference calculations were performed using DFT+CAS(8,8) (Complete Active Space).[3] Taking the DFT (PBE0) orbitals as starting point, we constructed the ab initio many-body Hamiltonian using the ORCA[4] software, which employs lib2int library to generate the kinetic and electrostatics integrals involving the orbitals that make up the active space (in this case, we choose the eight orbitals closest to Fermi Level and eight electrons). Once we have this integral file, we constructed the many-body Hamiltonian and diagonalized it exactly. Once the ground state was found, we constructed from it the 1-particle reduced density matrix[5] and obtained the natural orbitals (eigenfunctions) and the associated occupations (eigenvalues). An open-shell character was derived from occupations that depart significantly from 0 or 2, so that two single-occupied natural orbitals would correspond to a perfect diradical. This calculation yielded slightly different radical characters for different products. By using Density Matrix Renormalization Group[6] to solve the CAS-CI Hamiltonian on larger active spaces up to 18 orbitals, we checked that the results for natural orbitals converged and were reliable in the calculations with smaller active spaces.

We carried out dI/dV simulations of frontiers orbitals using PP-STM package[7] for a CO-like orbital tip, represented by a combination of P_xP_y (90%) and s-like wave character (10%) without tip relaxation.

Supplementary Tables, Schemes, and Figures

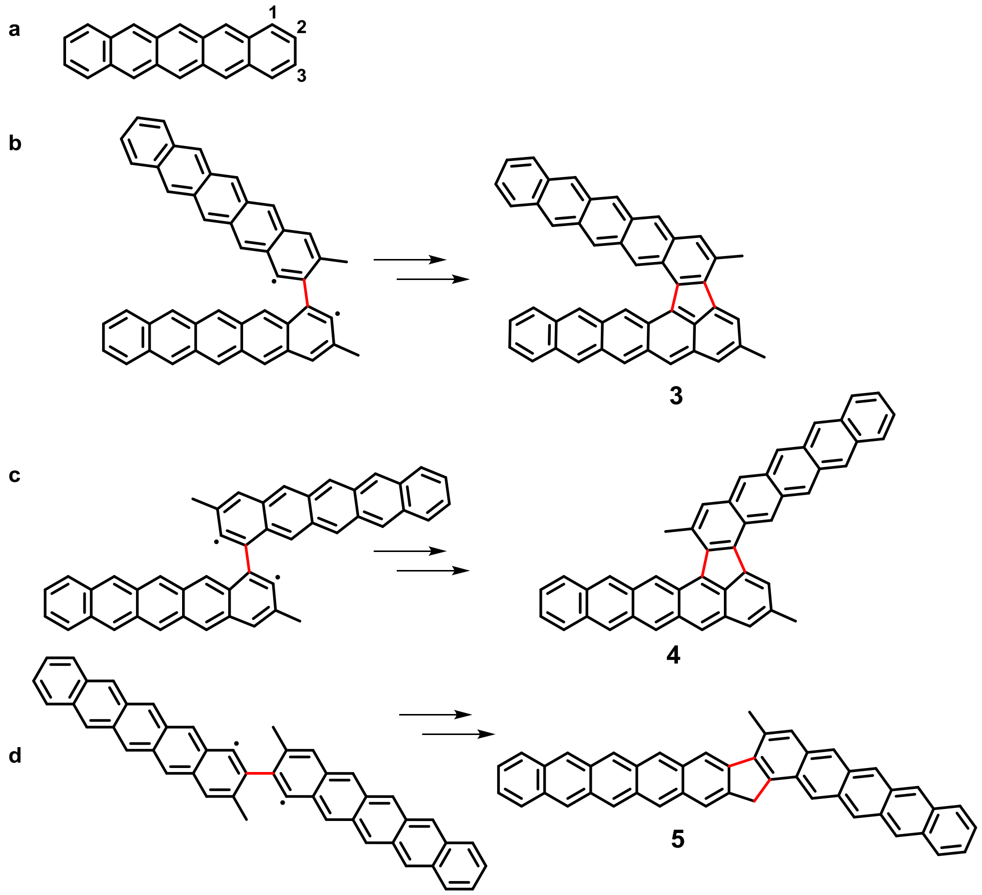

Scheme S1. (a) Structure of a pentacene with illustrated numbering of carbon atoms. (b-d) Schematic diagram of possible mono-coupling schemes followed by cyclodehydrogenations: (b) C1 of one pentacene unit to C2 of another; (c) C1 to C1 coupling; and (d) C2 to C2. We note the other radical will probably be quenched by the H released during cyclodehydrogenation step.[8] Halogens are not shown for clarity. Other products may be possible, but the steric hindrance due to the methyl group may prevent such pathways.

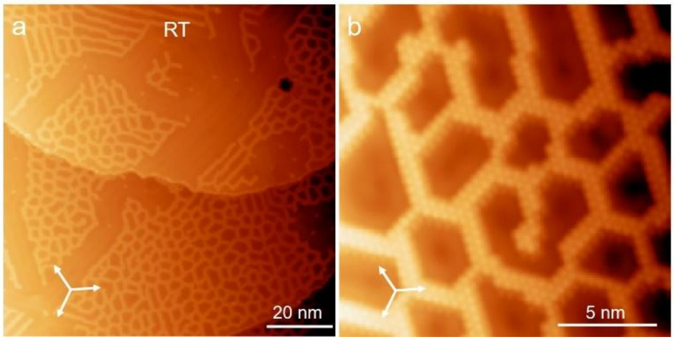

Figure S1. The sample prepared by depositing the precursor molecule on a Au(111) substrate at RT. Only Iodine adatoms can be observed on the surface, which self-assemble by √3×√3R30° with respect to the Au(111) lattice.[9] The three high symmetric direction of the Au(111) lattice are marked by white arrows. Scanning tunneling parameters: (a) U = 1 V, I = 100 pA; (b) U = 300 mV, I = 500 pA.

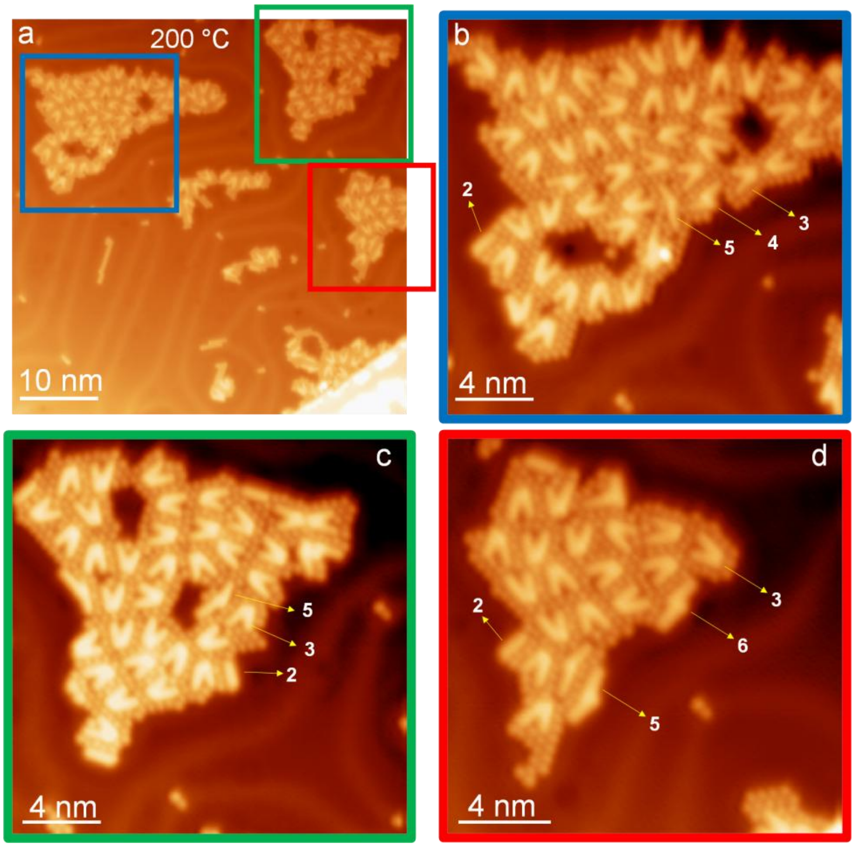

Figure S2. (a) Large scale image of a sample after molecule deposition on Au(111) held at 200ºC, marking molecular islands depicted in following zoom in images in panels (b), (c) and (d). The frames are colored accordingly and the images include marked examples for each of the molecular species as a guide to the reader.

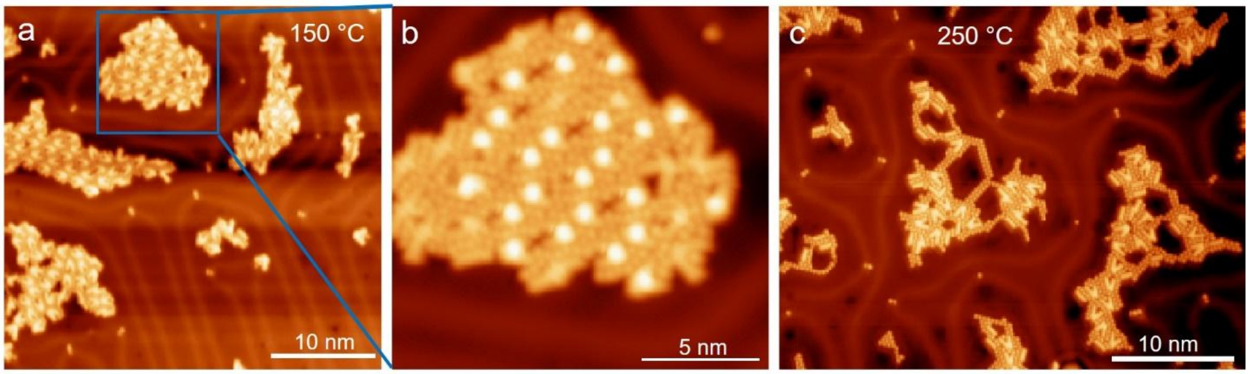

Figure S3. (a) Large-scale STM images of the sample prepared by depositing the precursor molecule on a Au(111) surface held at 150 °C. (b) Zoom-in STM image of the framed region in (a). The existence of bright protrusions implies most of carbonyl groups are still intact at this temperature. This is in consistence with previous report,[10] where decarbonylation reactions completed at 187 °C. Some products **6** can be observed at the edge of the self-assembled islands. More unassigned structures are observed on the sample prepared at 150 °C, which are presumably attributed to intermediates toward the thermodynamic products. (c) Large-scale STM image of the sample prepared by depositing the precursor molecule on a Au(111) surface held at 250 °C. Scanning parameters: (a) U = 1 V, I = 100 pA; (b) U = −800 mV, I = 100 pA; U = 500 mV, I = 50 pA.

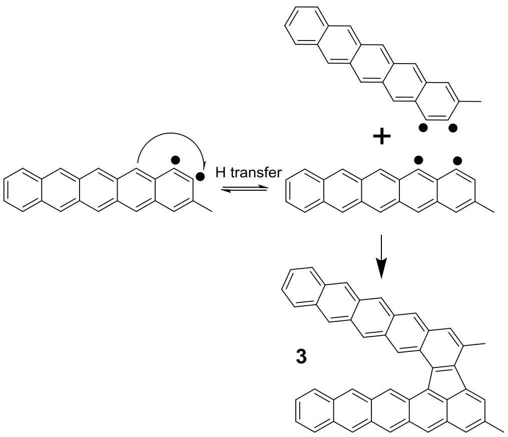

Figure S4. Schematic drawing of H-migration mechanism for the formation of dimer **3** (same for dimer **4**).

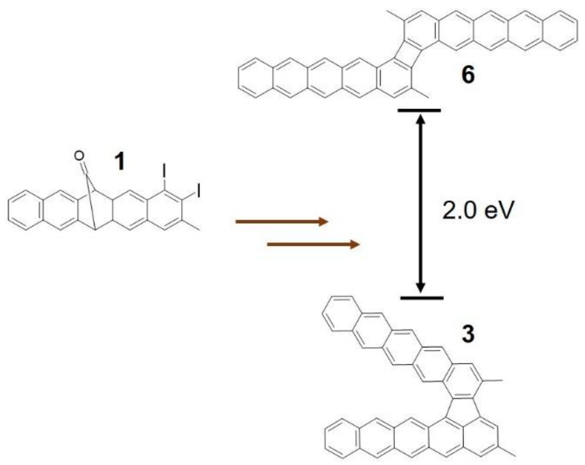

Figure S5. Schematic drawing showing dimer **6** has 2 eV higher total energy than dimer **3** on Au(111) according to DFT calculations.

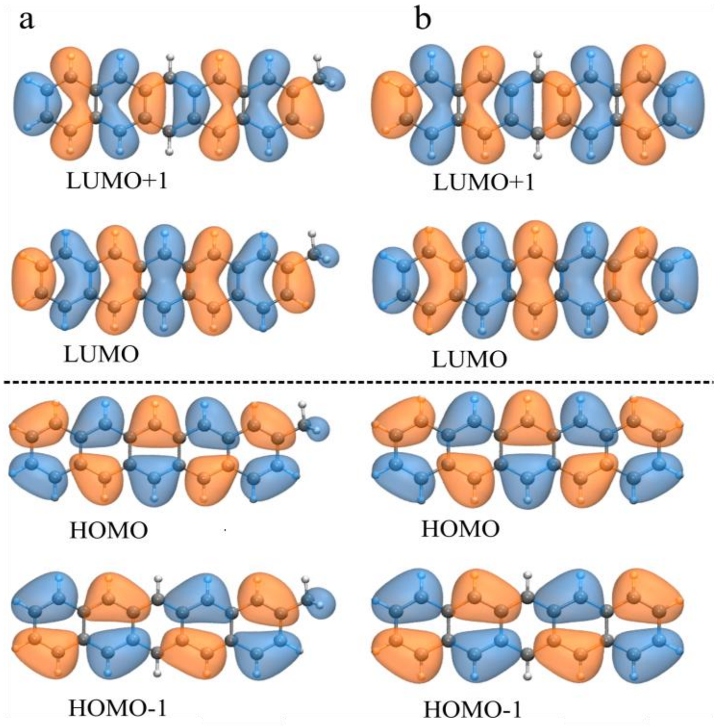

Figure S6. a) The spin-unrestricted DFT calculations for monomer **2** with the methyl functional yielded molecular orbitals for spin-α HOMO-1, HOMO, LUMO, and LUMO+1. The Fermi level is indicated by a dashed line. b) Monomer **2** without the methyl functional spin-unrestricted DFT calculations yielded

molecular orbitals for spin-α HOMO-1, HOMO, LUMO, and LUMO+1, with Fermi level indicated by a dashed line.

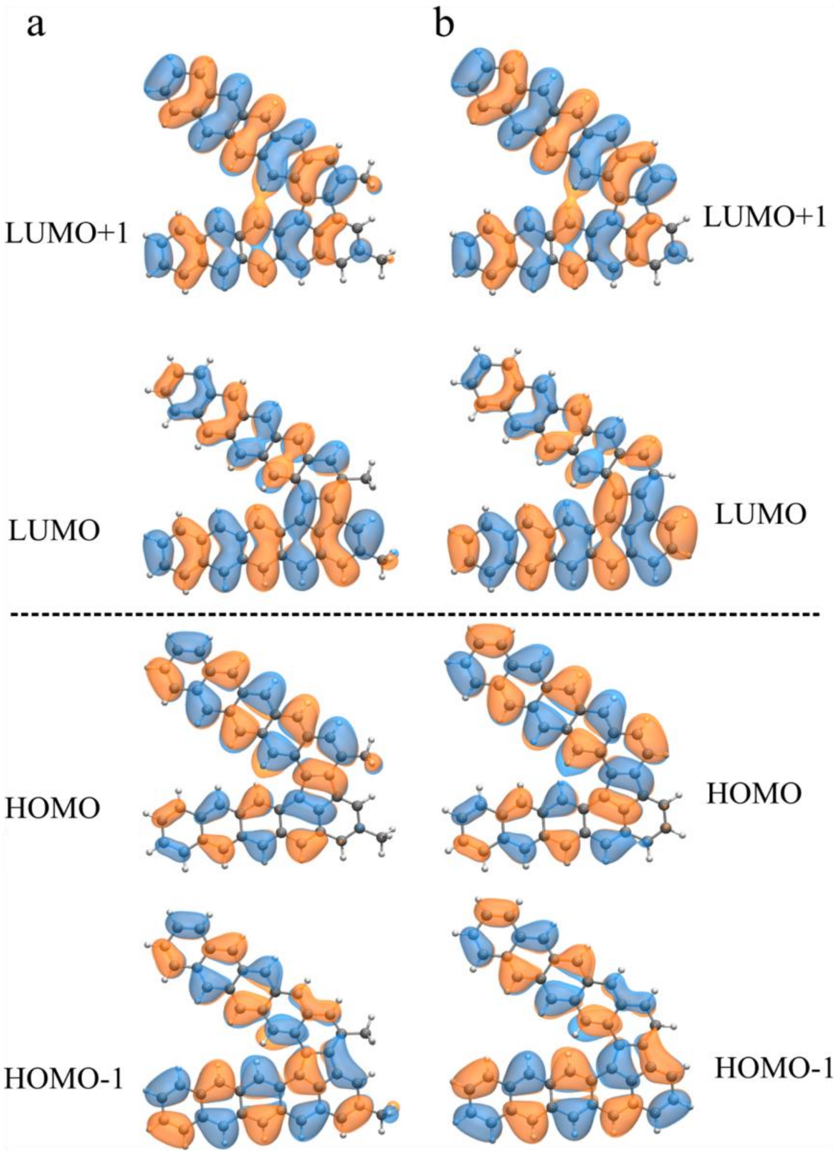

Figure S7. a) The spin-unrestricted DFT calculations for dimer **3** with the methyl functional yielded molecular orbitals for spin-α HOMO-1, HOMO, LUMO, and LUMO+1. The Fermi level is indicated by a dashed line. b) Dimer **3** without the methyl functional spin-unrestricted DFT calculations yielded molecular orbitals for spin-α HOMO-1, HOMO, LUMO, and LUMO+1, with Fermi level indicated by a dashed line.

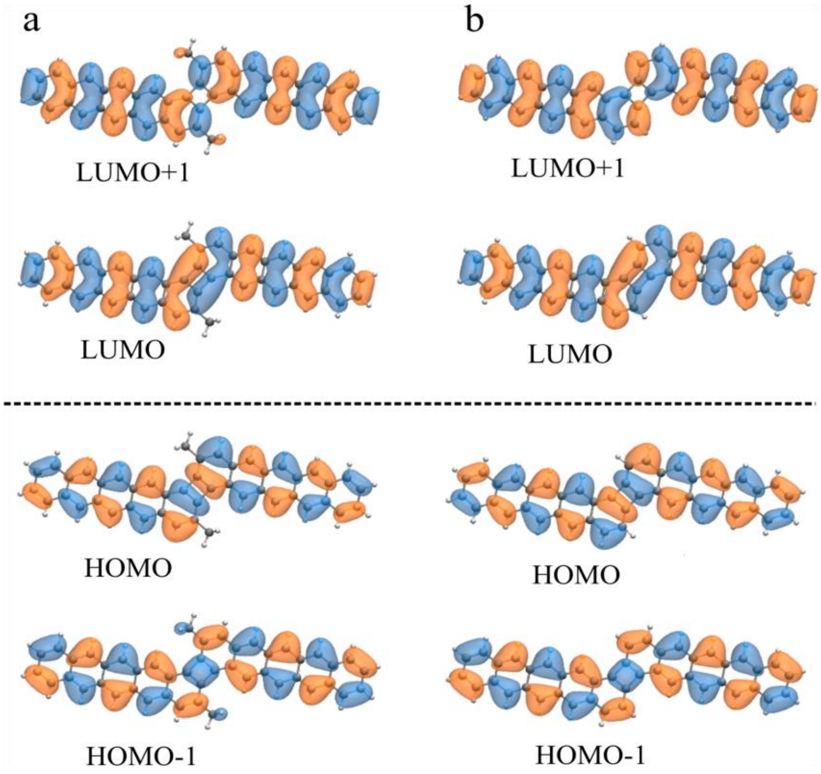

Figure S8. a) The spin-unrestricted DFT calculations for dimer 6 with the methyl functional yielded molecular orbitals for spin-α HOMO-1, HOMO, LUMO, and LUMO+1. The Fermi level is indicated by a dashed line. b) Dimer 6 without the methyl functional spin-unrestricted DFT calculations yielded molecular orbitals for spin-α HOMO-1, HOMO, LUMO, and LUMO+1, with Fermi level indicated by a dashed line.

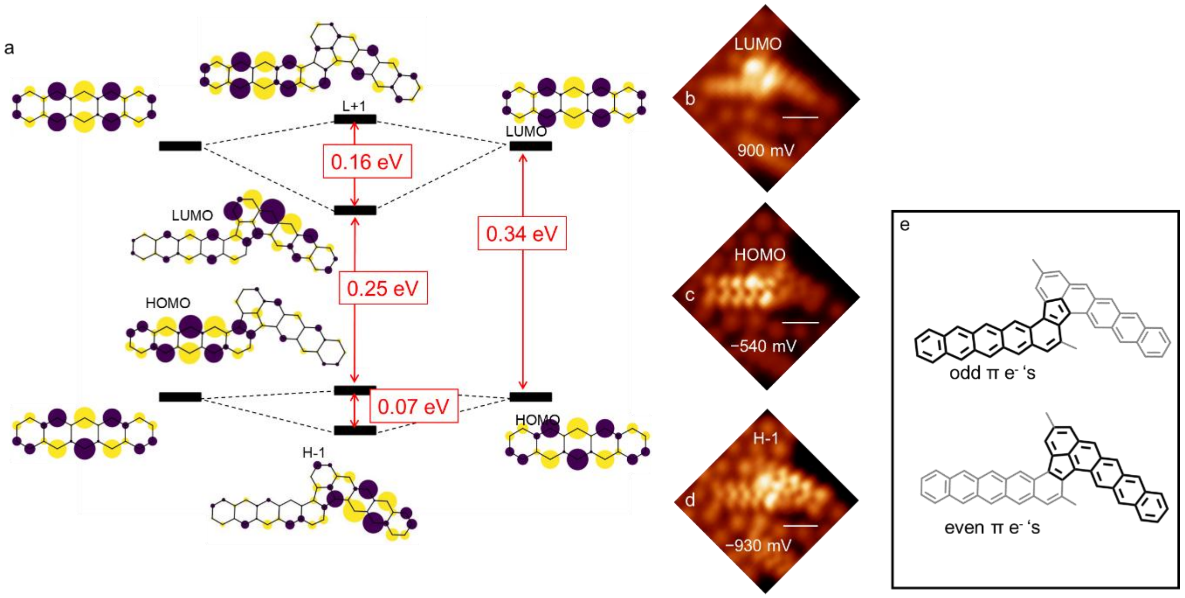

Figure S9. (a) Hückel orbitals of **4**. (b-d) Conductance maps for dimer product **4** taken at energies corresponding to LUMO, HOMO and HOMO-1. (e) Schematic partition of the dimer structure assigning the linking ring to one or another monomer. The combination with the even number of electrons hosts most of the bonding, lower energy orbital (HOMO-1), and the combination with the odd number of electrons hosts most of the anti-bonding, higher energy orbital (HOMO). All the scale bars are 5 Å.

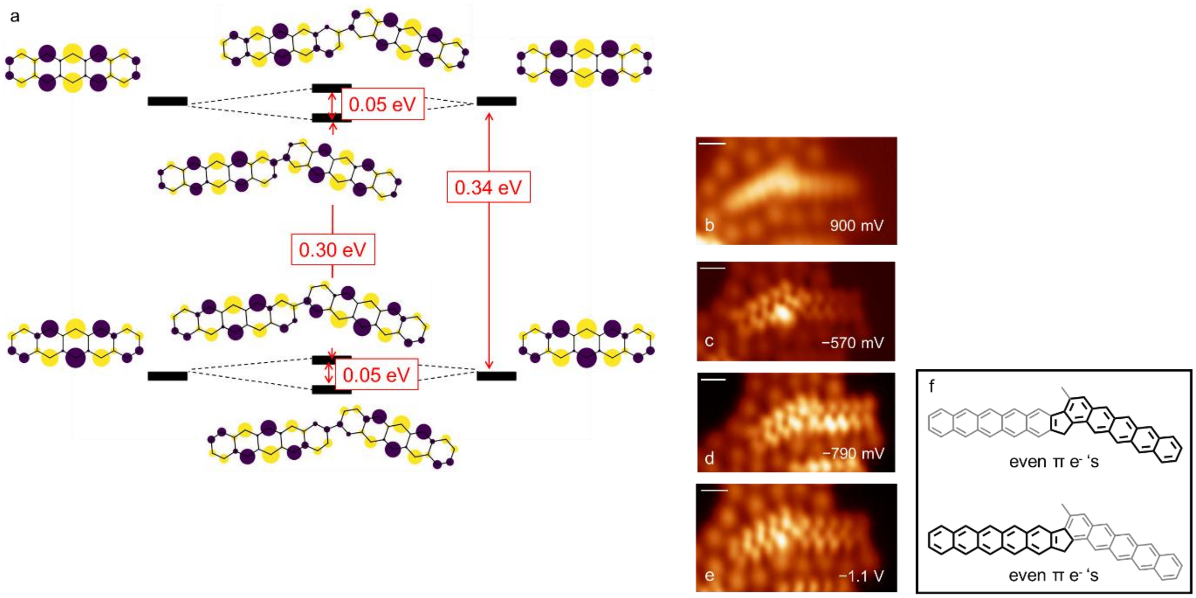

Figure S10. (a) Hückel orbitals of **5**. (b-e) Conductance maps for dimer product **5** taken at energies corresponding to HOMO-2, HOMO-1, HOMO, and LUMO. (f) Schematic partition of the dimer structure

assigning the linking ring to one or another monomer. Both two combinations have an even number of π electrons, thus the density of states of molecular orbitals more or less equally distribute over the two monomers. Note that there is a sp^3 hybridized carbon on the five-membered ring, which does not contribute to the π system of dimer **5**. One of the two hydrogens on this carbon atom can be removed by tip induced dehydrogenation procedure, as seen in the next figure. All the scale bars are 5 Å.

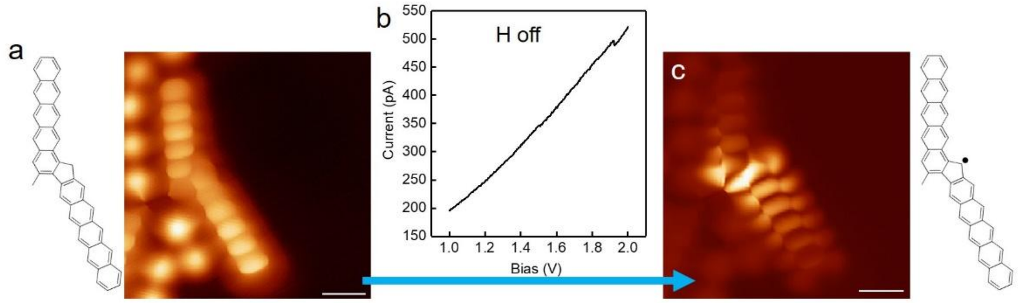

Figure S11. Tip induced dehydrogenation on dimer **5**. (a) Chemical structure and bond resolving STM image (recorded at 5 mV at constant height mode) of dimer **5** before dehydrogenation. (b) I-V curve recorded during dehydrogenation process. Tip was placed on the top of the sp^3 carbon and bias was ramped from 1 V to 2 V. A current jump can be observed around 1.9 V, which implies that the dehydrogenation occurred. (c) Bond resolving STM image (recorded at 5 mV at constant height mode) and chemical structure of dimer **5** after dehydrogenation. The five-membered ring looks smaller after dehydrogenation and low-energy radical states can be readily observed in the STM image, supporting the successful removal of the hydrogen atoms.[11–13] All the scale bars are 5 Å.

Table S1. Occupation numbers for the HONO and LUNO as obtained from DFT-CAS(8,8) and from DFT (PBE0) for products **2-6** and acenes of varying size for comparison.

**Molecule**	**HONO/LUNO**	
	**DFT-CAS**	**DFT PBE0**
2	1.86 / 0.14	2.0 / 0.0
3	1.91 / 0.09	1.97 / 0.03
4	1.91 / 0.1	1.93 / 0.07
5	1.9 / 0.1	1.98 / 0.02
6	1.77 / 0.23	1.58 / 0.42
Naphthalene	1.92 / 0.08	2.00 / 0.00
Anthracene	1.89 / 0.11	2.00 / 0.00
Tetracene	1.88 / 0.12	2.00 / 0.00
Pentacene	1.84 / 0.16	1.98 / 0.02
Hexacene	1.8 / 0.20	1.74 / 0.26
Heptacene	1.74 / 0.26	1.55 / 0.44

Table S2. Calculated NICS values of compounds **6** and **8**. Values were calculated on each ring starting from the four-membered ring outwards. Shown below the table is the reference for the numbering of each ring.

	Molecule 6				Molecule 8		
**Ring**	**NICS(0)**	**NICS(1)**	**NICS(1)$_{zz}$**	**Ring**	**NICS(0)**	**NICS(1)**	**NICS(1)$_{zz}$**
1	37.5	26.3	89.7	1	11.1	2.7	16.8
2	-7.1	-8.7	-20.4	2	-2.86	-5.0	-10.0
3	-8.9	-10.8	-27.5	3	-8.5	-10.5	-26.7
4	-9.8	-11.7	-30.1	4	-11.5	-13.0	-34.3
5	-8.7	-10.7	-27.2	5	-10.7	-12.5	-32.8
6	-4.5	-7.2	-17.9	6	-5.9	-8.5	-21.75

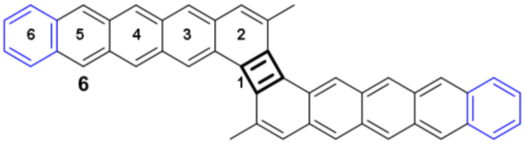

6

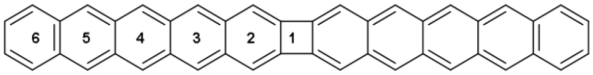

8

Precursor synthesis

Starting compounds, catalysts and solvents were purchased from Sigma-Aldrich and TCI. Flash column chromatography was performed by using silica gel (60 Å pore size, 40-63 µm Merck). The reactions were monitored by thin layer chromatography (TLC) on silica gel-coated plates (Merck 60 F_{254}). The NMR spectroscopic data in solution were recorded with Bruker Avance 300 MHz and 500 MHz instruments and were calibrated by using the residual undeuterated solvent as an internal reference (CD_2Cl_2 at δH = 5.33 ppm, δC = 53.84 ppm; $CDCl_3$ at δH = 7.26 ppm, δC = 77.16 ppm; tetrachloroethane-d_2 at δH = 6.00 ppm, δC = 73.78 ppm). Chemical shifts are reported in parts per million (ppm) on the δ scale and coupling constants (J) are in Hertz (Hz). The abbreviations used to describe the multiplicities are s = singlet, dd = doublet of doublets ddd = doublet of doublet of doublet. Mass spectra were recorded at the Service Commun de Spectrometrie de Masse of University Paul Sabatier (Toulouse 3), Toulouse (France).

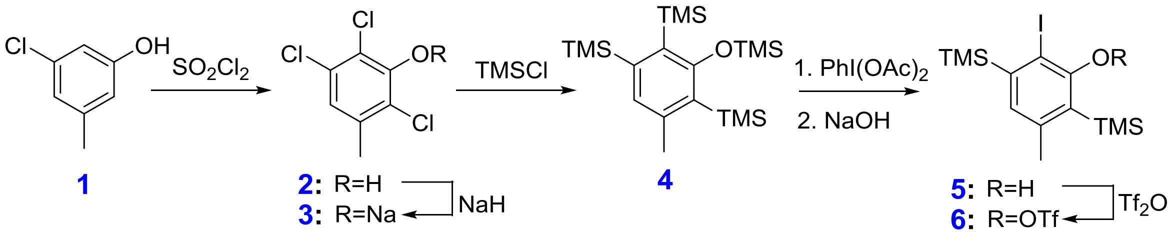

2,3,6-trichloro-5-methylphenol (**2**)

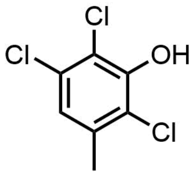

Commercially available 3-chloro-5-methylphenol **1** (10 g, 70 mmol) was dissolved in anhydrous toluene (800 ml) in a round-bottom flask equipped with a condenser. The solution was bubbled with an argon atmosphere for 10 min. Then anhydrous tetramethylpiperidine (2.4 ml, 14 mmol, 20 mol%) was added dropwise. Freshly distilled sulfuryl chloride (11.6 ml, 144 mmol, 2.05 eq.) was added dropwise and the reaction mixture was stirred under an argon atmosphere at 82 °C for 1.5 h. After cooling down, the reaction mixture was extracted with brine (2×300 ml) and the organic layer was dried over $MgSO_4$, then filtered through a paper filter and concentrated in vacuum providing the white product **2** (13.6 g, 92%) which was collected by filtration through the glass frit.

^{1}H NMR (300 MHz, $CDCl_3$): 2.33 (d, *J* = 0.7 Hz, 3H, CH_3), 5.97 (s, 1H, OH), 6.96 (d, *J* = 0.7 Hz, 1H).

^{13}C NMR (75 MHz, $CDCl_3$): 20.06 (CH_3), 117.40, 119.76, 123.03 (C-H), 130.96, 136.21, 148.88.

CI MS: ($[M+H]^+$) 211.

HR CI MS: calcd for $C_7H_6Cl_3O$ 210.9484; found 210.9478.

Sodium 2,3,6-trichloro-5-methylphenolate (**3**)

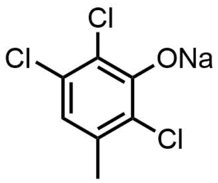

A round-bottom flask was charged with previous compound **2** (8.83 g, 41.8 mmol) and dissolved in anhydrous THF (150 ml). The solution was cooled with an ice bath while stirred under an argon atmosphere and a suspension of NaH (1.05 g, 44 mmol, 1.05 eq.) in anhydrous THF (40 ml) was carefully added dropwise to the reaction mixture via a cannula. The ice bath was removed and the reaction mixture was stirred under an argon atmosphere at room temperature for 1 h. The reaction mixture was evaporated *in vacuo* and properly dried providing the product **3** as a yellow powder (9.8 g, 99 %). This crude product was used directly in the next step, without further purification.

^{1}H NMR (300 MHz, acetone-d_6): 2.19 (d, J = 0.54 Hz, 3H, CH_3), 6.33 (d, J = 0.54 Hz, 1H).

^{13}C NMR (75 MHz, acetone-d_6): 20.31 (CH_3), 108.30 (C-H), 118.56, 122.34, 128.35, 133.23, 161.99.

CI MS: ($[M\text{-}Na+H]^+$) 211.

HR CI MS: calcd for $C_7H_6Cl_3O$ 210.9484; found 210.9482.

Trimethylsilyl 2,3,6-trimethylsilyl-5-methylphenolate (**4**)

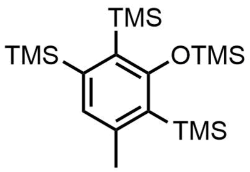

A Schlenk flask was charged with previous compound **3**, Mg powder (1.25 g, 51.4 mmol, 8.0 eq.), CuCl (0.64 g, 6.4 mmol, 1.0 eq.) and LiCl (2.2 g, 51.4 mmol). All components must be properly dried and of high qualities. The charged reaction flask was then evacuated and refiled with an argon atmosphere. Then highly pure anhydrous dimethylimidazolidone (23 ml) was added and the resulting suspension was degassed in a freeze-vacuum-thaw cycle (3×). Trimethylsilylchloride was then carefully added with a syringe, the reaction mixture was slowly heated at 55 °C under an argon atmosphere and rigorously stirred for 4 h. After cooling down, a resulting black dense reaction mixture was slowly poured to a cooled saturated $NaHCO_3$ solution (50 ml), then filtered through a glass frit S3 and the filtrate was extracted with hexane (3×25 ml). The combined organic layers were extracted with brine (2×50 ml) to remove a residuum of dimethylimidazolidone and dried over Na_2SO_4. After filtration through a paper filter, the organic solution was evaporated in vacuum, providing the crude product **4** as a brightly brown oil (2.15 g, 86%), which was used in the next reaction without its further purification.

^{1}H NMR (300 MHz, CD_2Cl_2): 0.09 (s, 9H, TMS), 0.29 (s, 9H, TMS), 0.31 (s, 9H, TMS), 0.32 (s, 9H, TMS), 2.37 (d, J = 0.54 Hz, 3H, CH_3), 6.89 – 7.09 (m, 1H).

^{13}C NMR (75 MHz, CD_2Cl_2): 1.08 (3C, TMS), 2.37 (3C, TMS), 2.78 (3C, TMS), 3.82 (3C, TMS), 23.11 (CH_3), 128.83, 131.66 (C-H), 134.45, 144.51, 148.96, 166.81.

CI MS: ($[M]^+$) 396.

HR CI MS: calcd for $C_{19}H_{40}OSi_4$ 396.2156; found 396.2154.

2-Iodo-3,6-tromethylsilyl-5-methyl-phenol (**5**)

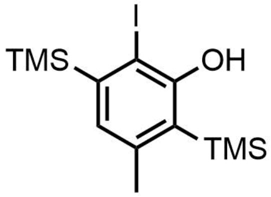

A previous compound **4** (5.50 g, 13.8 mmol) was dissolved in anhydrous dichloromethane (70 ml) in a Schlenk flask under an argon atmosphere. This solution was cooled down at -50 °C with a dry ice – ethanol bath. In another Schlenk flask was prepared solution of I_2 (1.76 g, 6.9 mmol, 0.50 eq.) and $PhI(OAc)_2$ (2.23 g, 6.9 mmol, 0.50 eq.) in anhydrous dichloromethane (30 ml). The resulting deep purple solution was stirred under an argon atmosphere for 30 min to properly dissolve all the components then this solution was then transferred to the first cooled solution of **4** via a cannula. The cooling bath was removed and the reaction mixture was left to warm up to room temperature and continued with stirring for another 1 h. The reaction mixture was reduced under vacuum to 1/3 of its initial volume and then poured to a solution of KOH (0.86 g, 15.3 mmol, 1.10 eq.) in methanol (40 ml). After stirring this reaction mixture under an argon atmosphere at room temperature for 30 min., the reaction mixture was then evaporated in vacuum. The solid residuum was poured in to water (100 ml) and extracted with ethyl acetate (3×40 ml). The combined organic layers were finally extracted with a saturated aqueous solution of $Na_2S_2O_7$ (100 ml) and brine (100 ml), then dried over Na_2SO_4. Chromatography on a silica gel column (hexane) provided the product **5** as a colorless oil (1.56 g, 30%).

^{1}H NMR (300 MHz, CD_2Cl_2): 0.34 (s, 9H, TMS), 0.38 (s, 9H, TMS), 2.34 (d, J = 0.6 Hz, 3H, CH_3), 5.72 (s, 1H, OH), 6.70 (d, J = 0.6 Hz, 1H).

^{13}C NMR (75 MHz, CD_2Cl_2): -0.34 (3C, TMS), 2.16 (3C, TMS), 23.81 (CH_3), 94.51 (C-I), 131.56, 145.56 (2C), 147.67, 158.13.

CI MS: ($[M]^+$) 378.

HR CI MS: calcd for $C_{13}H_{23}IOSi_2$ 378.0332; found 378.0338.

2-iodo-5-methyl-3,6-bis(trimethylsilyl)phenyl trifluoromethanesulfonate (6)

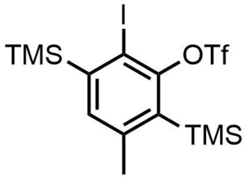

In a well dry Schlenk flask was dissolved previous compound **5** (0.5 g, 1.30 mmol) in anhydrous dichloromethane (3 ml). Then anhydrous pyridine (0.65 ml, 6.15 mmol, 5.0 eq.) was added and the reaction mixture was stirred under an argon atmosphere at room temperature for 15 min. Then trifluoromethansulfonic anhydride (0.7 ml, 3.9 mmol, 3.0 eq.) was added and the reaction mixture was heated at 50 °C and stirred under an argon atmosphere for 1.5 h. The reaction mixture was then evaporated in vacuum and purified by column chromatography (SiO_2, hexane), providing the product **6** as a colorless oil (0.22 g, 33%).

^{1}H NMR (300 MHz, CD_2Cl_2): 0.41 (s, 9H, TMS, a), 0.43 (s, 9H, TMS), 2.46 (d, J = 0.5 Hz, 2H, CH_3), 7.05 – 7.34 (m, 1H, aromatic H).

^{13}C NMR (75 MHz, CD_2Cl_2): 0.04, 1.86, 23.53, 95.84, 119.08 (q, J = 320.8 Hz, Tf), 136.87, 138.64, 146.61, 150.68, 151.39.

^{19}F NMR (282 MHz, CD_2Cl_2): -71.92.

CI MS: ($[M]^+$) 510.

HR CI MS: calcd for $C_{14}H_{22}F_3IO_3SSi_2$ 509.9825; found 509.9801.

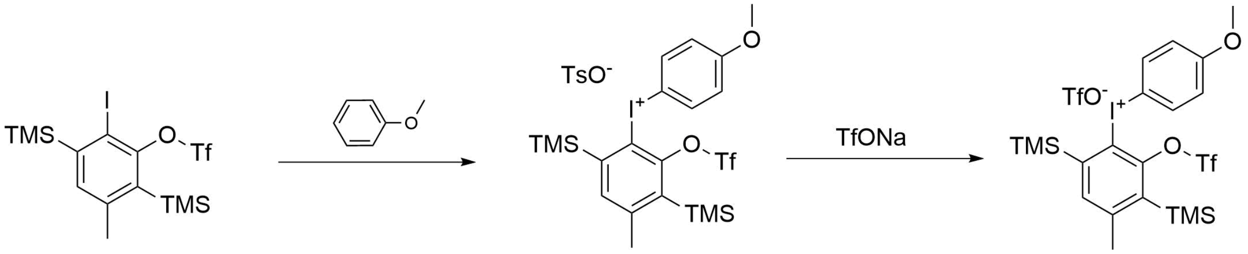

6 7a 7b

In a dry round bottom flask **6** (850 mg, 1.67 mmol) was dissolved in DCM (21 mL) and trifluoroethanol (21 mL). To this solution *p*-toluenesulfonic acid monohydrate (634 mg, 3.33 mmol, 2 equiv.) was added followed by mCPBA (570 mg, 3.33 mmol, 2 equiv.) and the reaction mixture was stirred at room temperature for 1 hour. Then, anisole (272 µL, 2.5 mmol, 1.5 equiv.) was added and the reaction was stirred for another 20 hours. The reaction mixture was evaporated and purified by chromatography on reverse phase silica gel (gradient hexane to hexane : EtOAc 1:1) to get product **7a** contaminated with tosic acid. The product **7a** (1.14 g) was dissolved in DCM (23 mL) and a solution of TfONa (4.0 g, 23 mmol, 15equiv.) in water (23 mL) was added. This two-phase mixture was vigorously stirred for 1 hour. Then, organic phase was collected and the water phase was extracted with DCM. All gathered organic fractions were dried over $MgSO_4$. After evaporation of the solvent, the residue was purified by column chromatography (SiO_2, DCM/MeOH 20:1) to get **7b** (716 mg, 56%) as a yellowish amorphous solid.

^{1}H NMR (500 MHz, CD_2Cl_2): 0.39 (9H, s, TMS), 0.47 (9H, s, TMS), 2.59 (3H, s, CH_3), 3.82 (3H, s, OCH_3), 6.92 – 6.97 (2H, m), 7.44 (1H, d, J = 0.6 Hz), 7.61 – 7.66 (2H, m).

^{13}C NMR (126 MHz, CD_2Cl_2): 0.67 (TMS), 1.52 (TMS), 24.21 (CH_3), 56.44 (OCH_3), 106.10, 117.85, 118.38 (2C), 118.95 (q, J = 321.0 Hz, Tf), 121.06 (q, J = 320.2 Hz, Tf), 135.37 (2C), 140.05, 140.21, 147.97, 152.38, 153.10, 163.19.

^{19}F NMR (282 MHz, CD_2Cl_2): -79.01, -71.84.

ESI MS: 617 ($[M]^+$).

ESI MS: 149 ($[M]^-$).

HR ESI MS: calcd for $C_{21}H_{29}F_3IO_4SSi_2^+$ 617.0316; found 617.0338.

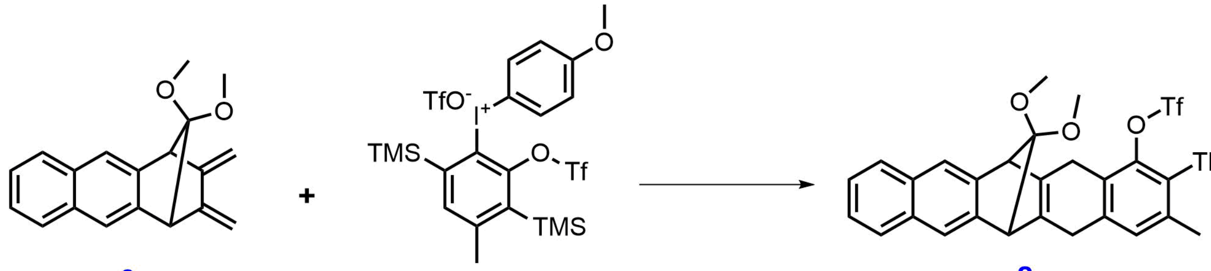

(6S)-15,15-dimethoxy-3-methyl-2-(trimethylsilyl)-5,6,13,14-tetrahydro-6,13-methanopentacen-1-yl trifluoromethanesulfonate 8

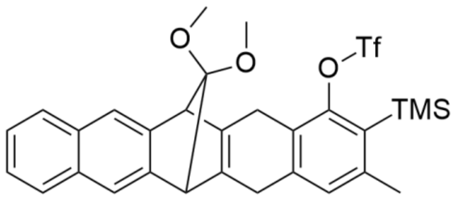

A well-dried Schlenk flask was charged with diene **9** (140 mg, 0.50 mmol, 1.0 equiv.) and hypervalent iodine **7b** (424 mg, 0.553 mmol, 1.1 equiv.) under argon and then anhydrous acetonitrile (15 mL) was added. The mixture was cooled to 0°C and then CsF (168 mg, 1.1 mmol, 2.2 equiv.) was added in one portion. The mixture was stirred at 0°C for 10 min. After this time, the solvent was evaporated and the residue was purified by column chromatography (SiO_2, hexane/EtOAc 10:1) to get **8** as a white amorphous solid (165 mg, 56%).

^{1}H NMR (500 MHz, CD_2Cl_2): 0.36 (9H, s, TMS), 2.41 (3H, s, CH_3), 3.08 (3H, s, OCH_3), 3.29 (3H, s, OCH_3), 3.30-3.40 (2H, m), 3.67-3.72 (2H, m), 4.00 (2H, s), 7.01 (1H, s), 7.37-7.40 (2H, m), 7.59 (1H, s), 7.61 (1H, s), 7.69-7.73 (1H, m).

^{13}C NMR (126 MHz, CD_2Cl_2): 1.92 (TMS), 23.57 (CH_3), 25.07 (CH_2), 29.83 (CH_2), 50.99 (CH_3O), 52.31 (CH_3O), 56.54, 56.78, 119.16 (q, J = 319.15, Tf), 119.72, 119.80, 125.78 (2C), 126.05, 126.16, 128.00, 128.06, 131.44, 131.83, 132.74 (2C), 138.69, 139.70, 140.22, 144.62, 145.39, 145.53, 150.54.

^{19}F NMR (282 MHz, CD_2Cl_2): -73.88.

DCI MS: 589 ($[M+H]^+$).

HR DCI MS: calcd for $C_{30}H_{31}F_3O_5SSi$ 588.1614; found 588.1603.

(13R)-15,15-dimethoxy-3-methyl-2-(trimethylsilyl)-6,13-dihydro-6,13-methanopentacen-1-yl trifluoromethanesulfonate 10

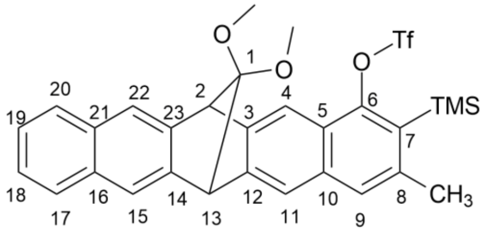

In a well dried flask, **8** (184 mg, 0.312 mmol, 1.0 equiv.) was dissolved in anhydrous toluene (19 mL) and then DDQ (106 mg, 0.469 mmol, 1.5 equiv.) was added in one portion. The reaction was stirred at room temperature for 20 hours. The reaction was filtered over glass frit S3 and the solvent was

removed under reduced pressure and the residue was purified by column chromatography (SiO_2, hexane/dichloromethane 1:1) to get **10** as a white amorphous solid (153 mg, 84%).

^{1}H NMR (500 MHz, CD_2Cl_2): 0.46 (9H, s, TMS), 2.59 (3H, s, CH_3), 3.22 (3H, s, OCH_3), 3.22 (3H, s, OCH_3), 4.73 (2H, s, *H2*, *H13*), 7.38 – 7.41 (2H, m, *H18*, *H19*), 7.55 (1H, s, *H9*), 7.65 (1H, s, *H11*), 7.73 (1H, s, *H15*), 7.73 (1H, s, *H22*), 7.72 – 7.75 (2H, m, *H17*, *H20*), 7.83 (1H, s, *H4*).
^{13}C NMR (126 MHz, CD_2Cl_2): 2.10 (TMS), 24.00 (CH_3), 51.55 (CH_3O), 51.61 (CH_3O), 55.32 (*C13*), 55.56 (*C2*), 114.39 (*C4*), 119.33 (q, J = 320.5 Hz, Tf), 119.88 (*C11*), 121.05 (*C15*), 121.16 (*C22*), 124.50 (*C5*), 125.48 (*C1*), 126.12 (*C18*, *C19*), 128.18 (*C17*), 128.26 (*C20*), 129.24 (*C9*), 131.76 (*C7*), 133.09 (*C16*), 133.13 (*C21*) 135.09 (*C10*), 141.26 (*C8*), 143.16 (*C14*), 143.46 (*C23*), 144.76 (*C3*), 145.88 (*C12*), 149.20 (*C6*).
^{19}F NMR (282 MHz, CD_2Cl_2): -72.81.
DCI MS: 587 ($[M+H]^+$).
HR DCI MS: calcd for $C_{30}H_{29}F_3O_5SSi$ 586.1457; found 586.1450.

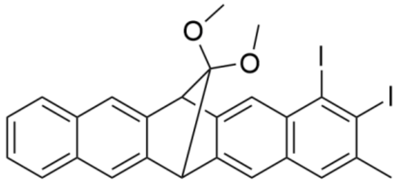

(6R)-1,2-diiodo-15,15-dimethoxy-3-methyl-6,13-dihydro-6,13-methanopentacene 11

A well dried flask was charged with **10** (50 mg, 85.2 µmol), CsF (51.8 mg, 0.341 mmol, 4 equiv.) and I_2 (44.3 mg, 0.170 mmol, 2 equiv.) under argon and then anhydrous CH_3CN was added (4 mL). The reaction mixture was then heated at 90°C for 2 days. The reaction mixture was evaporated and the residue was purified by column chromatography (SiO_2, hexane/$CHCl_3$ 2:3) to obtain **11** as a white amorphous solid (35 mg, 66%).

^{1}H NMR (500 MHz, CD_2Cl_2): 2.71 (3H, s), 3.21 (3H, s), 3.22 (3H, s), 4.73 (1H, s), 4.77 (1H, s), 7.37-7.41 (2H, m), 7.52 (1H, s), 7.64 (1H, s), 7.71-7.75 (2H, m), 7.71 (1H, s), 7.75 (1H, s), 8.14 (1H, s).

^{13}C NMR (126 MHz, CD_2Cl_2): 33.54, 51.61 (2C), 55.07, 55.40, 115.68, 116.83, 120.73, 121.10, 121.23, 125.54, 126.12, 126.13, 128.17, 128.22, 128.80, 129.09, 133.04, 133.06, 133.39, 134.86, 140.52, 143.23, 143.43, 145.24, 146.59,

DCI MS: 619 ($[M+H]^+$).
HR DCI MS: calcd for $C_{26}H_{20}I_2O_2$ 617.9553; found 617.9550.

(6R)-1,2-diiodo-3-methyl-6,13-dihydro-6,13-methanopentacen-15-one 12

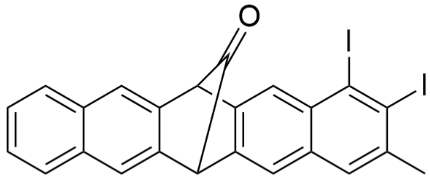

In a well dried Schlenk flask, **11** (25 mg, 40.44 µmol) was dissolved in anhydrous dichloromethane (3 mL) under argon. Then trimethylsilyl iodide (5.8 µL, 40.44 µmol, 1 equiv.) was added dropwise and the homogeneous reaction mixture was stirred overnight at room temperature. Next day the heterogeneous reaction mixture was stirred on air at room temperature for 6 hours to complete the hydrolysis of formed iodo-methoxy intermediate. The reaction mixture was evaporated and the residue was sonicated in a mixture of solvents (hexane : Et_2O, 2:1). The product **12** was collected by filtration over a glass frit filter S4 as a white solid (22.7 mg, 98%).

^{1}H NMR (300 MHz, Tetrachloroethane-d_2): 2.75 (3H, s), 5.01 (1H, s), 5.07 (1H, s), 7.46-7.52 (2H, m), 7.71 (1H, s), 7.74 (1H, s), 7.80-7.86 (2H, m), 7.95 (1H, s), 7.99 (1H, s), 8.37 (1H, s).

^{13}C NMR (126 MHz, Tetrachloroethane-d_2): 33.23, 56.56, 56.85, 115.59, 117.67, 120.51, 120.85, 120.99, 126.24, 126.25, 127.78, 127.81, 128.07, 128.91, 132.43 (2C), 132.69, 134.23, 136.46, 136.66, 138.40, 139.62, 140.48, 192.44.

DCI MS: 544 ($[M-CO]^+$).
HR DCI MS: calcd for $C_{23}H_{14}I_2$ 543.9185; found 543.9168.

References

(1) Geuenich, D.; Hess, K.; Köhler, F.; Herges, R. Anisotropy of the Induced Current Density (ACID), a General Method To Quantify and Visualize Electronic Delocalization. *Chem. Rev.* **2005**, *105* (10), 3758–3772. https://doi.org/10.1021/cr0300901.

(2) Frisch, M. J.; Trucks, G. W.; Schlegel, H. B.; Scuseria, G. E.; Robb, M. A.; Cheeseman, J. R.; Scalmani, G.; Barone, V.; Petersson, G. A.; Nakatsuji, H.; Li, X.; Caricato, M.; Marenich, A. V.; Bloino, J.; Janesko, B. G.; Gomperts, R.; Mennucci, B.; Hratchian, H. P.; Ortiz, J. V.; Izmaylov, A. F.; Sonnenberg, J. L.; Williams; Ding, F.; Lipparini, F.; Egidi, F.; Goings, J.; Peng, B.; Petrone, A.; Henderson, T.; Ranasinghe, D.; Zakrzewski, V. G.; Gao, J.; Rega, N.; Zheng, G.; Liang, W.; Hada, M.; Ehara, M.; Toyota, K.; Fukuda, R.; Hasegawa, J.; Ishida, M.; Nakajima, T.; Honda, Y.; Kitao, O.; Nakai, H.; Vreven, T.; Throssell, K.; Montgomery Jr., J. A.; Peralta, J. E.; Ogliaro, F.; Bearpark, M. J.; Heyd, J. J.; Brothers, E. N.; Kudin, K. N.; Staroverov, V. N.; Keith, T. A.; Kobayashi, R.; Normand, J.; Raghavachari, K.; Rendell, A. P.; Burant, J. C.; Iyengar, S. S.; Tomasi, J.; Cossi, M.; Millam, J. M.; Klene, M.; Adamo, C.; Cammi, R.; Ochterski, J. W.; Martin, R. L.; Morokuma, K.; Farkas, O.; Foresman, J. B.; Fox, D. J. Gaussian 16 Rev. C.01, 2016.

(3) Roos, B. O.; Taylor, P. R.; Sigbahn, P. E. M. A Complete Active Space SCF Method (CASSCF) Using a Density Matrix Formulated Super-CI Approach. *Chem. Phys.* **1980**, *48* (2), 157–173. https://doi.org/10.1016/0301-0104(80)80045-0.

(4) Neese, F. The ORCA Program System. *WIREs Comput. Mol. Sci.* **2012**, *2* (1), 73–78. https://doi.org/10.1002/wcms.81.

(5) Löwdin, P.-O. Quantum Theory of Many-Particle Systems. I. Physical Interpretations by Means of Density Matrices, Natural Spin-Orbitals, and Convergence Problems in the Method of Configurational Interaction. *Phys. Rev.* **1955**, *97* (6), 1474–1489. https://doi.org/10.1103/PhysRev.97.1474.

(6) Brabec, J.; Brandejs, J.; Kowalski, K.; Xantheas, S.; Legeza, Ö.; Veis, L. Massively Parallel Quantum Chemical Density Matrix Renormalization Group Method. **2020**. https://doi.org/10.48550/ARXIV.2001.04890.

(7) Krejčí, O.; Hapala, P.; Ondráček, M.; Jelínek, P. Principles and Simulations of High-Resolution STM Imaging with a Flexible Tip Apex. *Phys. Rev. B* **2017**, *95* (4), 045407. https://doi.org/10.1103/PhysRevB.95.045407.

(8) González-Herrero, H.; Mendieta-Moreno, J. I.; Edalatmanesh, S.; Santos, J.; Martín, N.; Écija, D.; de la Torre, B.; Jelinek, P. Atomic Scale Control and Visualization of Topological Quantum Phase Transition in π-Conjugated Polymers Driven by Their Length. *Adv. Mater.* **2021**, *33* (44), 2104495. https://doi.org/10.1002/adma.202104495.

(9) Huang, L.; Zeppenfeld, P.; Horch, S.; Comsa, G. Determination of Iodine Adlayer Structures on Au(111) by Scanning Tunneling Microscopy. *J. Chem. Phys.* **1997**, *107* (2), 585–591. https://doi.org/10.1063/1.474419.

(10) Zugermeier, M.; Gruber, M.; Schmid, M.; Klein, B. P.; Ruppenthal, L.; Müller, P.; Einholz, R.; Hieringer, W.; Berndt, R.; Bettinger, H. F.; Gottfried, J. M. On-Surface Synthesis of Heptacene and Its Interaction with a Metal Surface. *Nanoscale* **2017**, *9* (34), 12461–12469. https://doi.org/10.1039/C7NR04157H.

(11) Li, J.; Sanz, S.; Castro-Esteban, J.; Vilas-Varela, M.; Friedrich, N.; Frederiksen, T.; Peña, D.; Pascual, J. I. Uncovering the Triplet Ground State of Triangular Graphene Nanoflakes Engineered with

Atomic Precision on a Metal Surface. *Phys. Rev. Lett.* **2020**, *124* (17), 177201. https://doi.org/10.1103/PhysRevLett.124.177201.

(12) Wang, T.; Berdonces-Layunta, A.; Friedrich, N.; Vilas-Varela, M.; Calupitan, J. P.; Pascual, J. I.; Peña, D.; Casanova, D.; Corso, M.; de Oteyza, D. G. Aza-Triangulene: On-Surface Synthesis and Electronic and Magnetic Properties. *J. Am. Chem. Soc.* **2022**, *144* (10), 4522–4529. https://doi.org/10.1021/jacs.1c12618.

(13) Berdonces-Layunta, A.; Lawrence, J.; Edalatmanesh, S.; Castro-Esteban, J.; Wang, T.; Mohammed, M. S. G.; Colazzo, L.; Peña, D.; Jelínek, P.; de Oteyza, D. G. Chemical Stability of (3,1)-Chiral Graphene Nanoribbons. *ACS Nano* **2021**, *15* (3), 5610–5617. https://doi.org/10.1021/acsnano.1c00695.

^{1}H and ^{13}C NMR spectra of 2 – 12

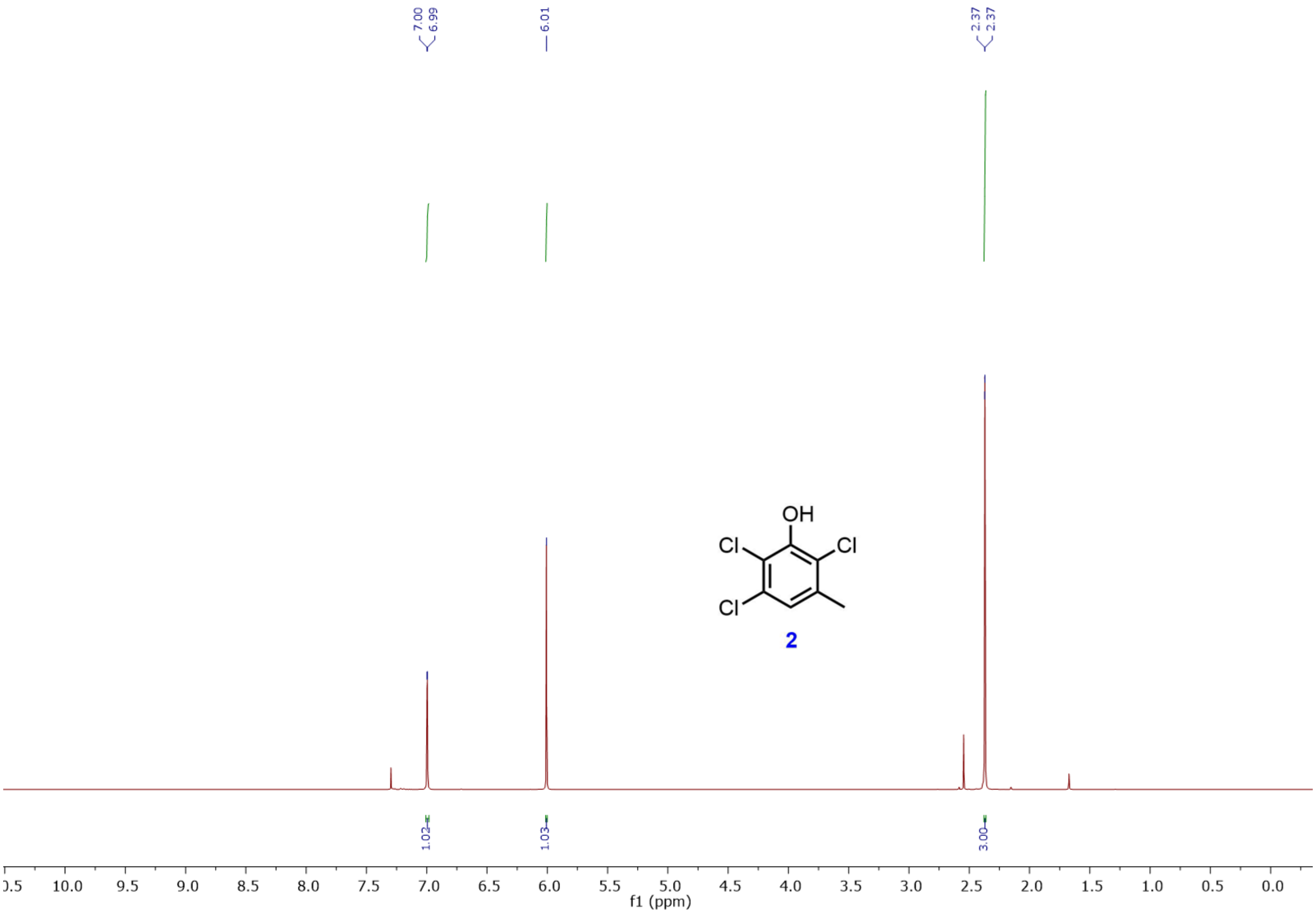

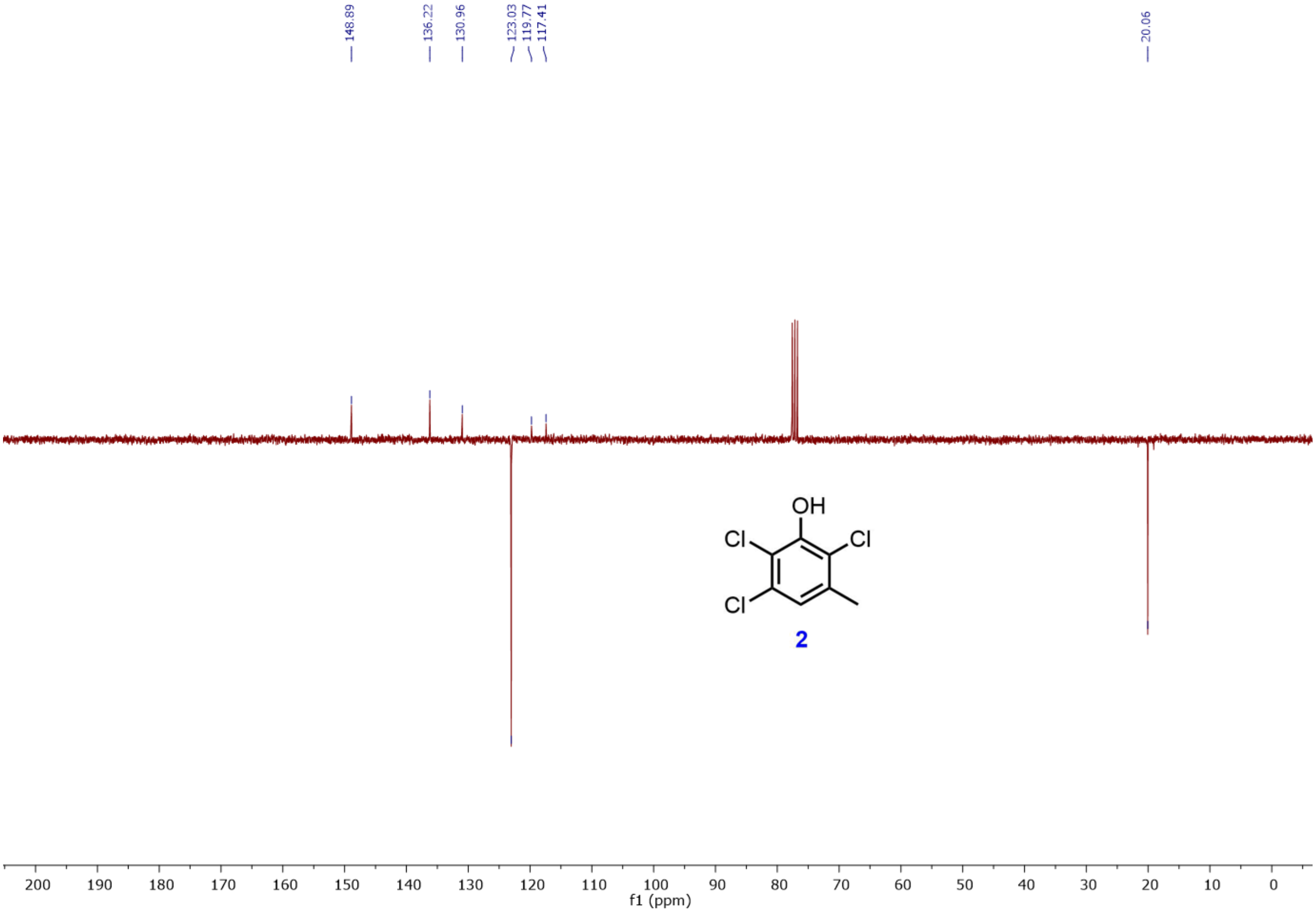

148.89
136.22
130.96
123.03
119.77
117.41
20.06
OH
Cl
Cl
Cl
2
200 190 180 170 160 150 140 130 120 110 100 90 80 70 60 50 40 30 20 10 0
f1 (ppm)

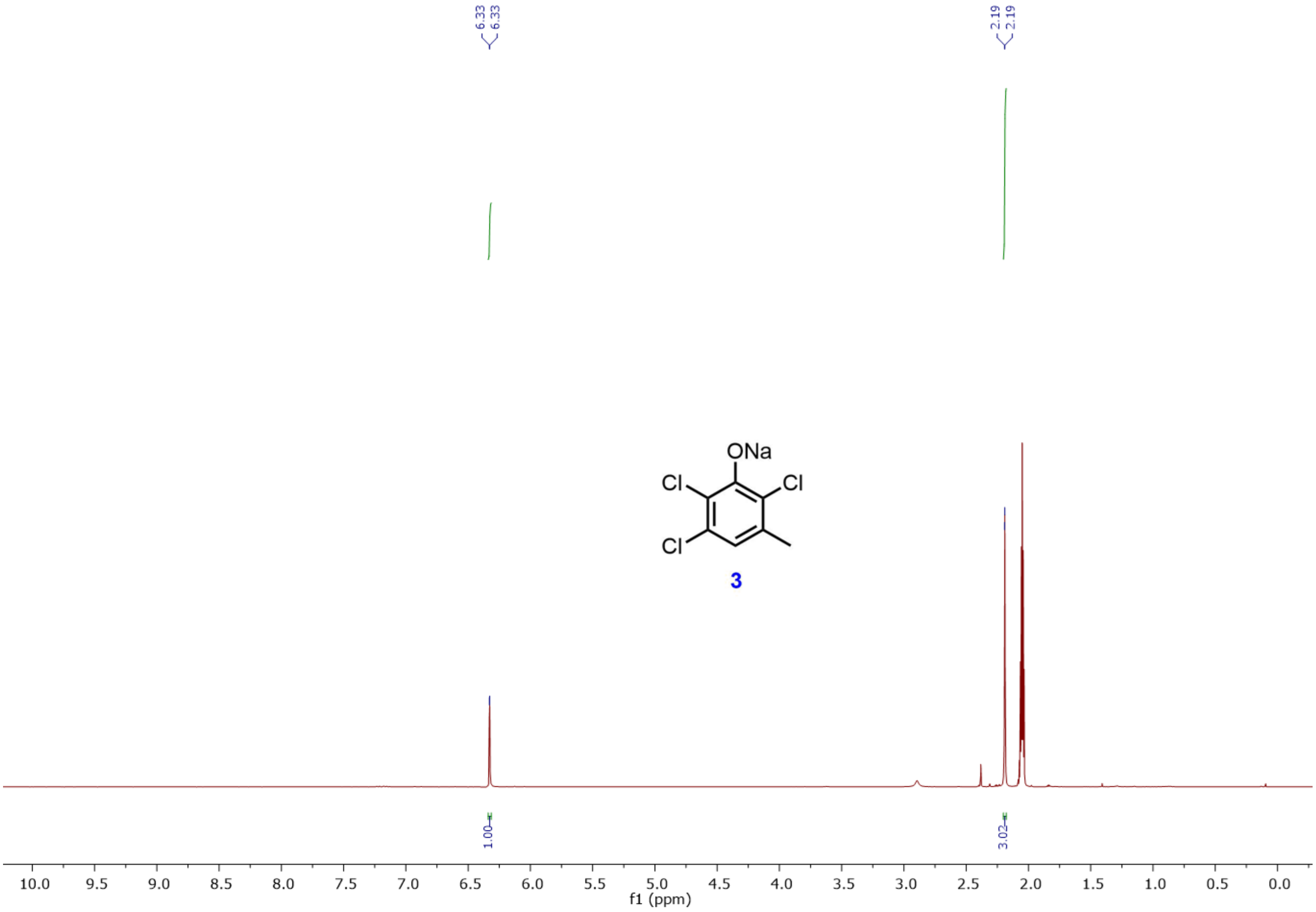
6.33
6.33
2.19
2.19
ONa
Cl
Cl
Cl
3
1.00
3.02
10.0 9.5 9.0 8.5 8.0 7.5 7.0 6.5 6.0 5.5 5.0 4.5 4.0 3.5 3.0 2.5 2.0 1.5 1.0 0.5 0.0
f1 (ppm)

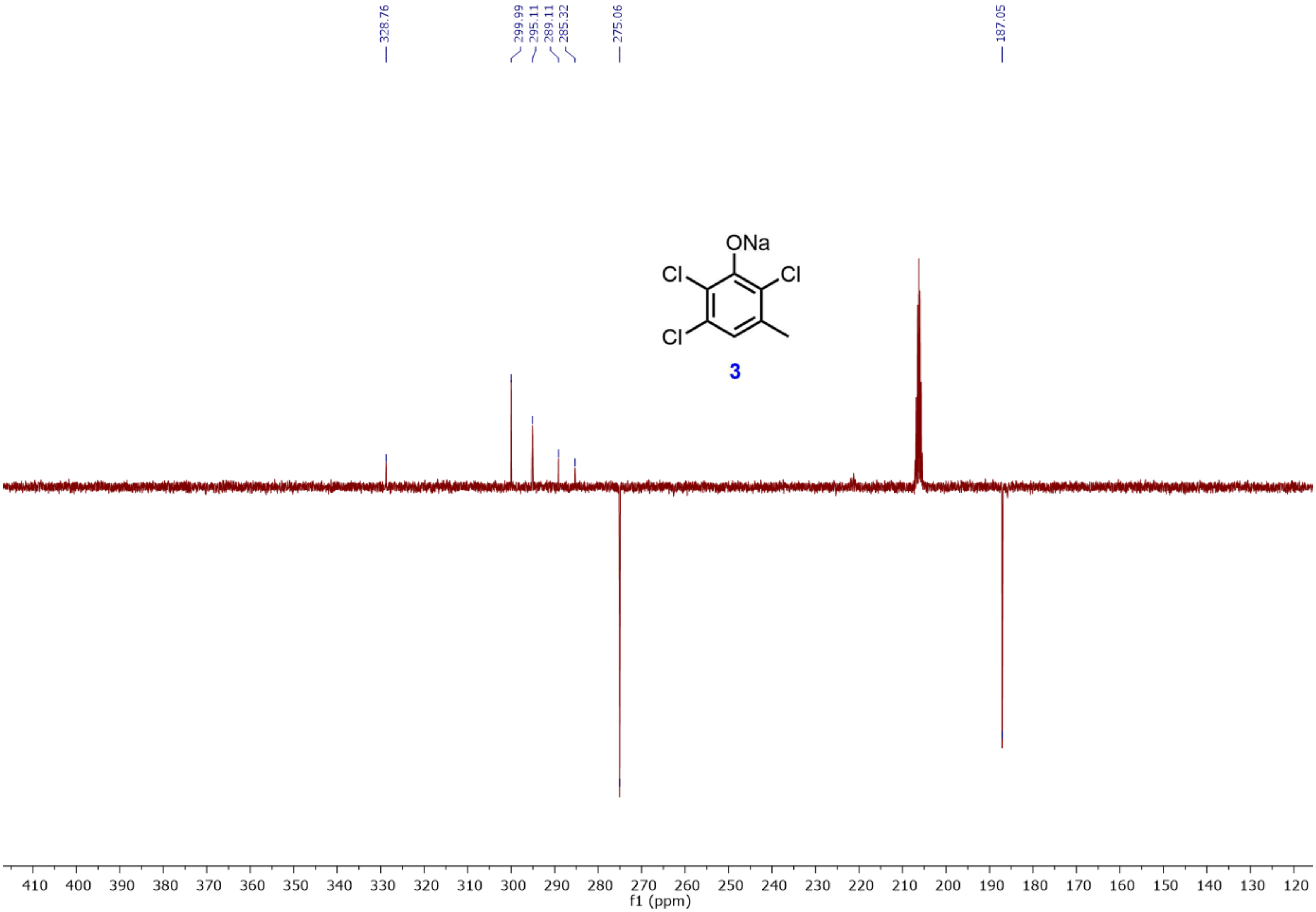

328.76
299.99
295.11
289.11
285.32
275.06
187.05
ONa
Cl
Cl
Cl
3
410 400 390 380 370 360 350 340 330 320 310 300 290 280 270 260 250 240 230 220 210 200 190 180 170 160 150 140 130 120
f1 (ppm)

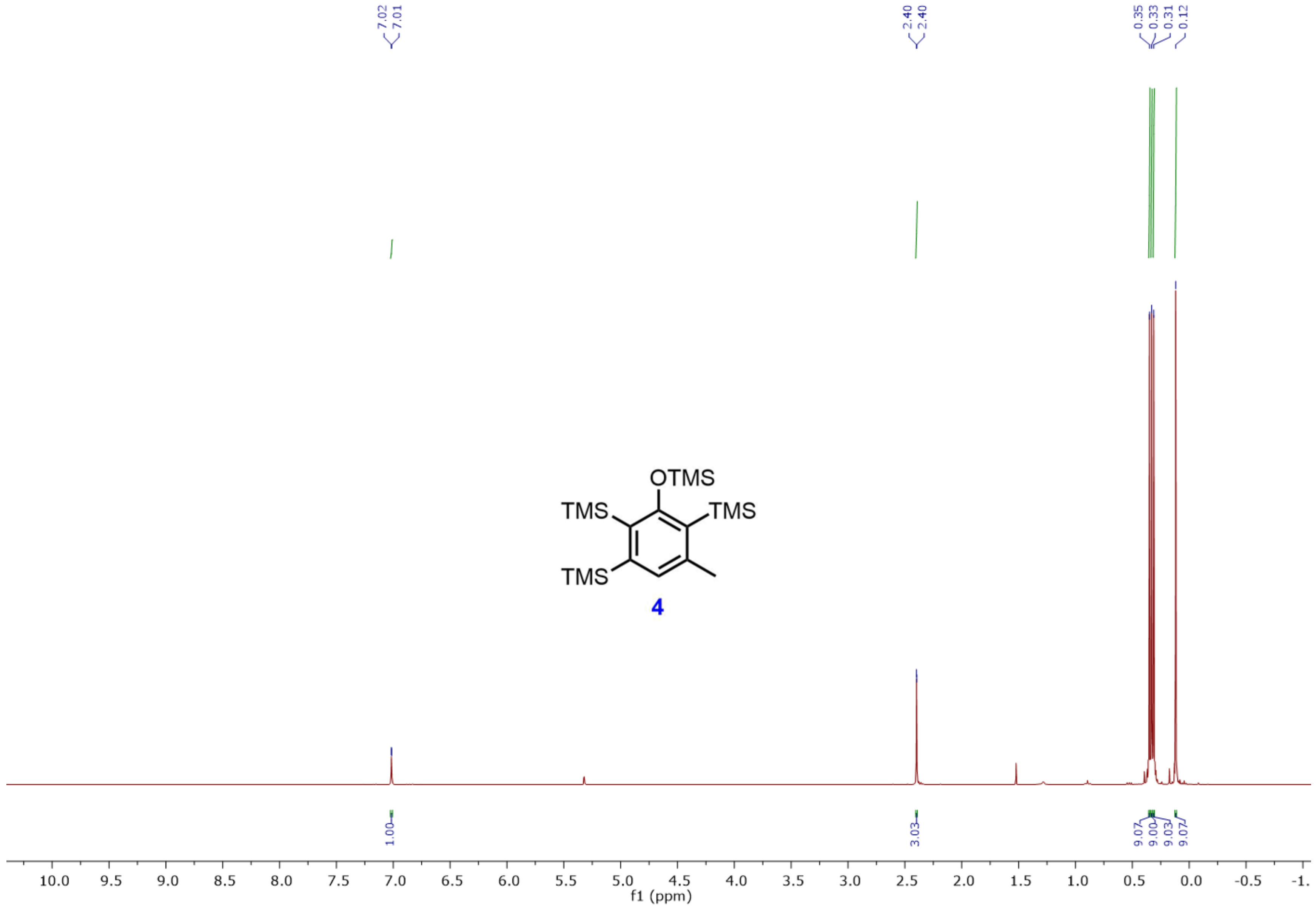
7.02
7.01
2.40
2.40
0.35
0.33
0.31
0.12
OTMS
TMS
TMS
TMS
4
1.00
3.03
9.07
9.00
9.03
9.07
10.0 9.5 9.0 8.5 8.0 7.5 7.0 6.5 6.0 5.5 5.0 4.5 4.0 3.5 3.0 2.5 2.0 1.5 1.0 0.5 0.0 -0.5 -1.
f1 (ppm)

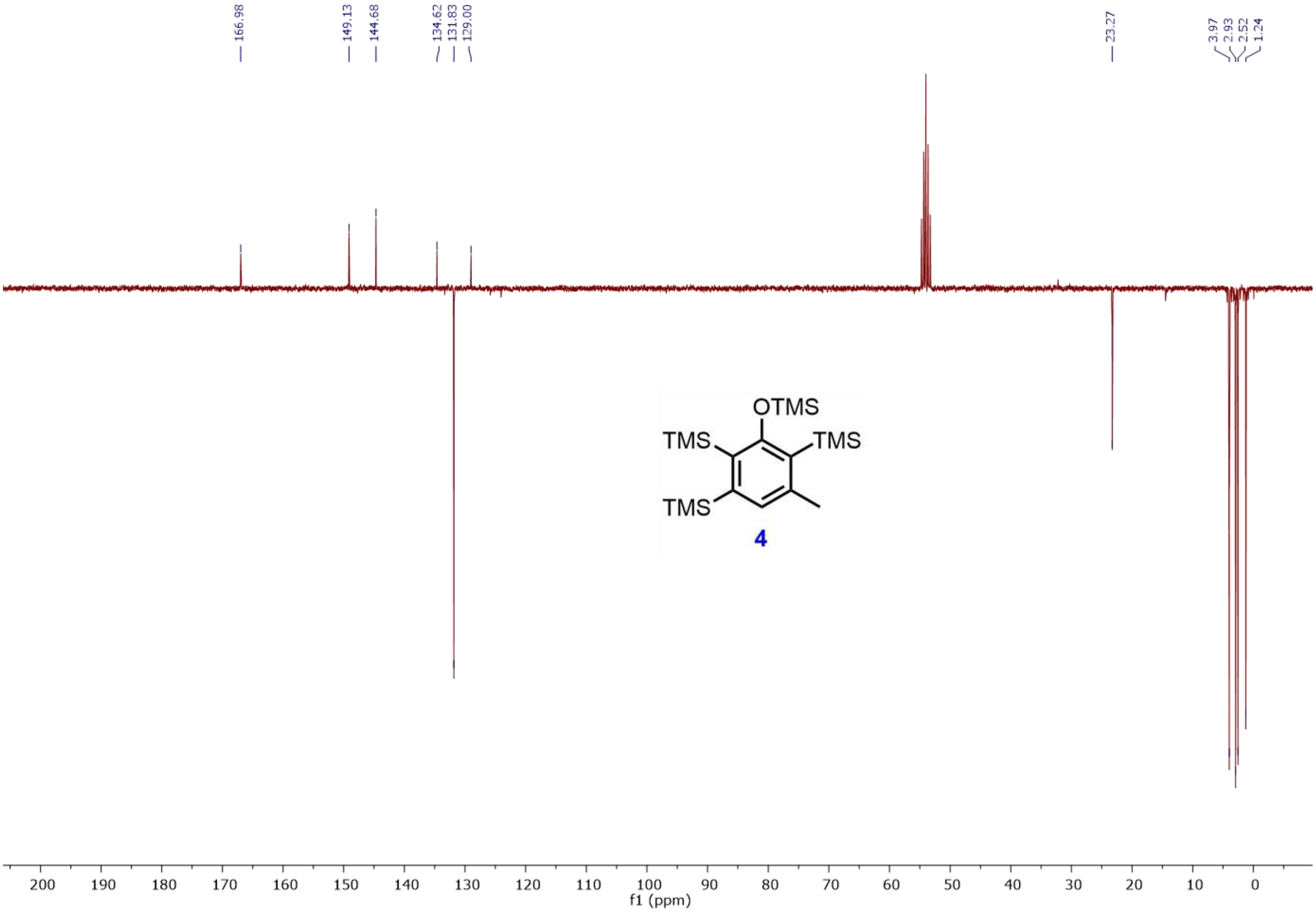

166.98
149.13
144.68
134.62
131.83
129.00
23.27
3.97
2.93
2.52
1.24
OTMS
TMS
TMS
TMS
4
200 190 180 170 160 150 140 130 120 110 100 90 80 70 60 50 40 30 20 10 0
f1 (ppm)

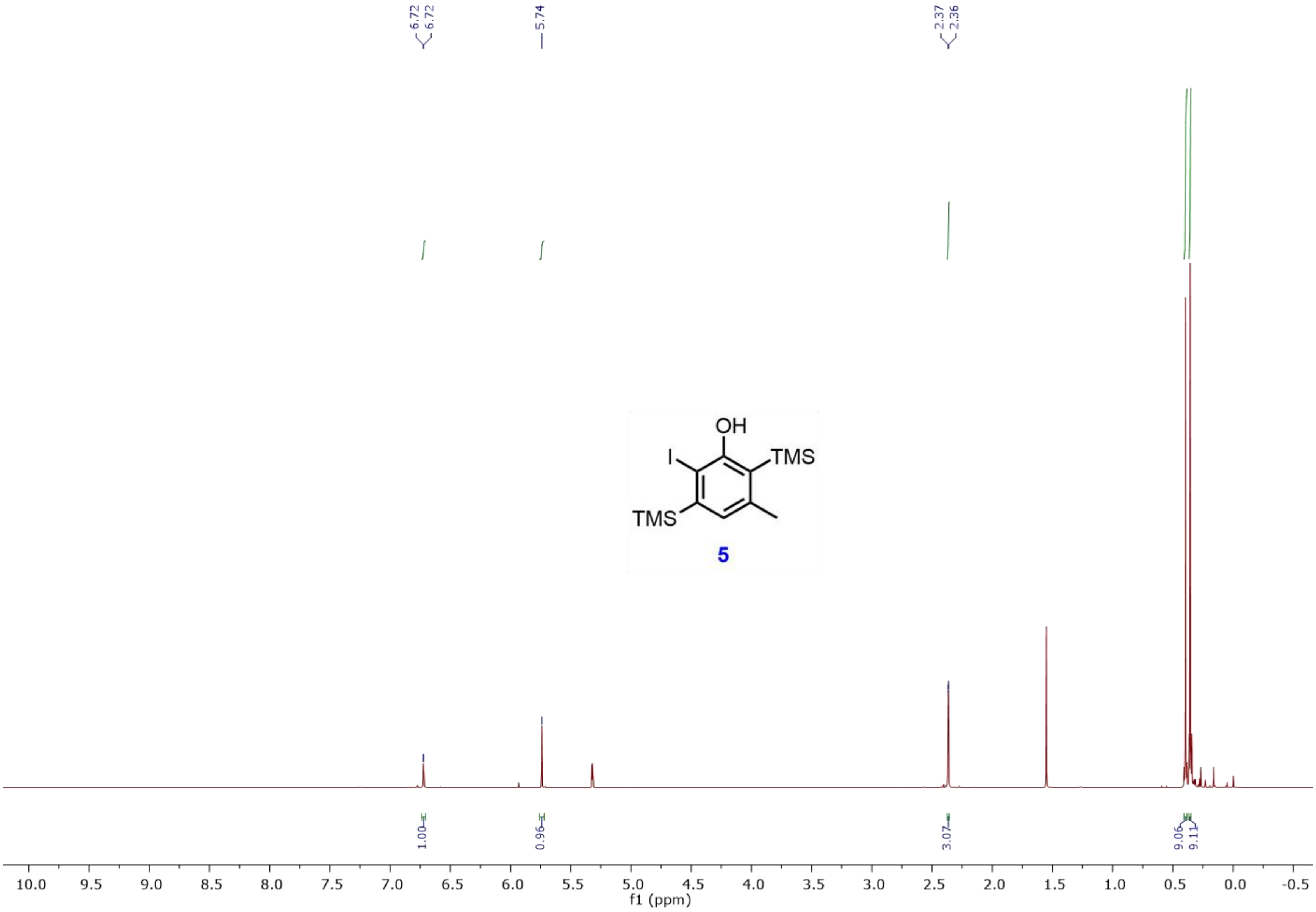
6.72
6.72
5.74
2.37
2.36
OH
I
TMS
TMS
5
1.00
0.96
3.07
9.06
9.11
10.0 9.5 9.0 8.5 8.0 7.5 7.0 6.5 6.0 5.5 5.0 4.5 4.0 3.5 3.0 2.5 2.0 1.5 1.0 0.5 0.0 -0.5
f1 (ppm)

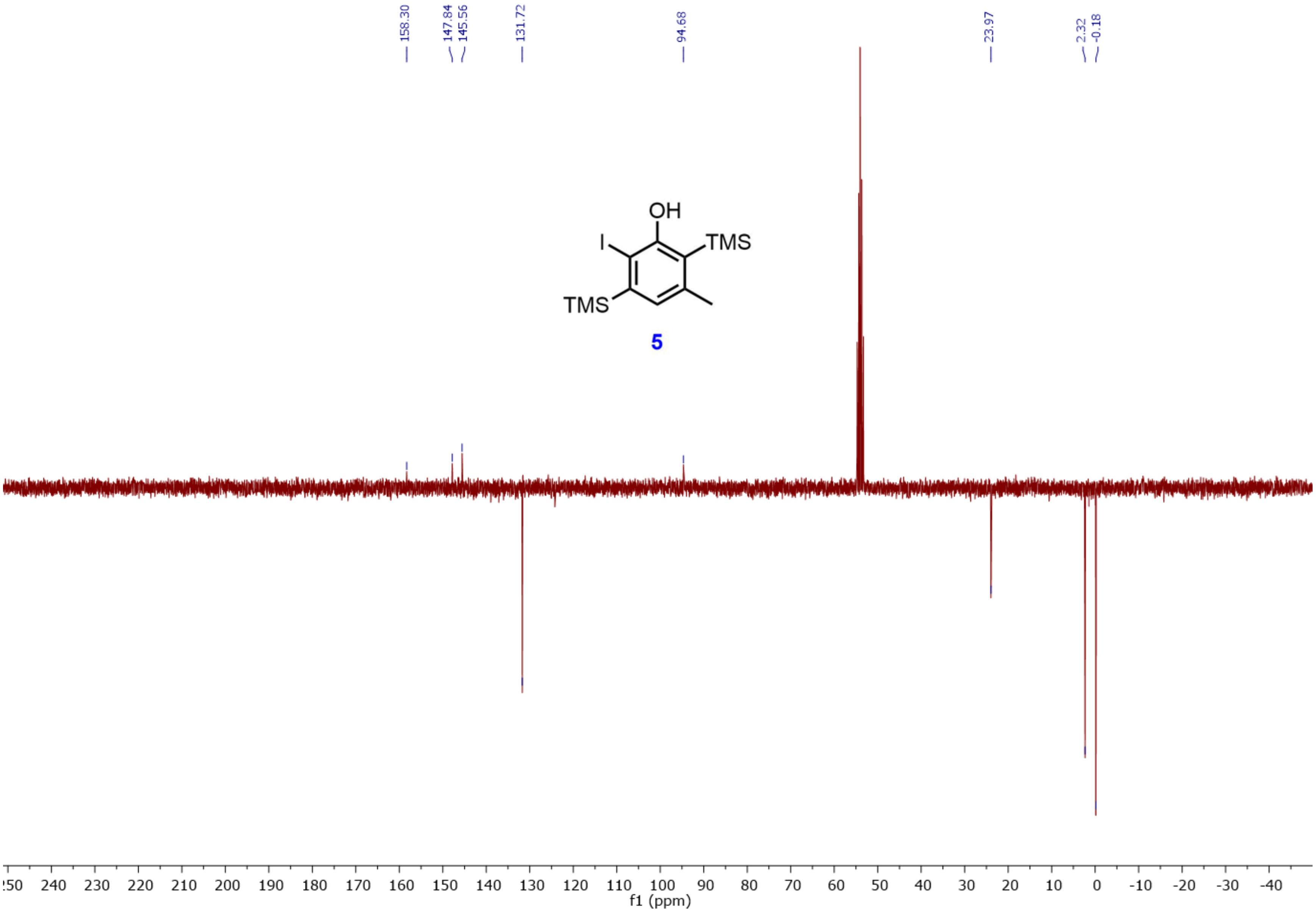
158.30
147.84
145.56
131.72
94.68
23.97
2.32
-0.18
OH
I
TMS
TMS
5
250 240 230 220 210 200 190 180 170 160 150 140 130 120 110 100 90 80 70 60 50 40 30 20 10 0 -10 -20 -30 -40
f1 (ppm)

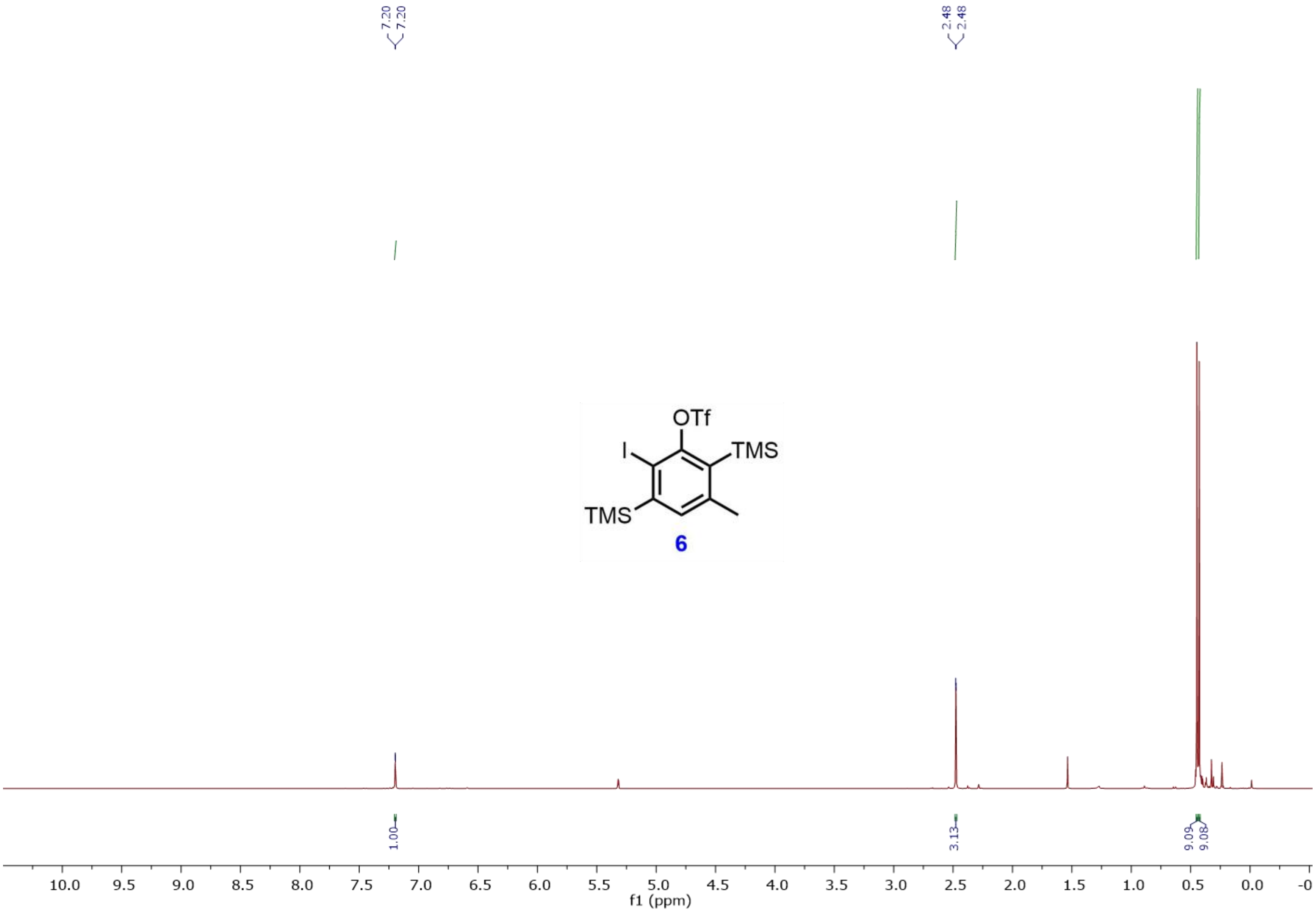
7.20
7.20
2.48
2.48
OTf
I
TMS
TMS
6
1.00
3.13
9.09
9.08
10.0 9.5 9.0 8.5 8.0 7.5 7.0 6.5 6.0 5.5 5.0 4.5 4.0 3.5 3.0 2.5 2.0 1.5 1.0 0.5 0.0 -0
f1 (ppm)

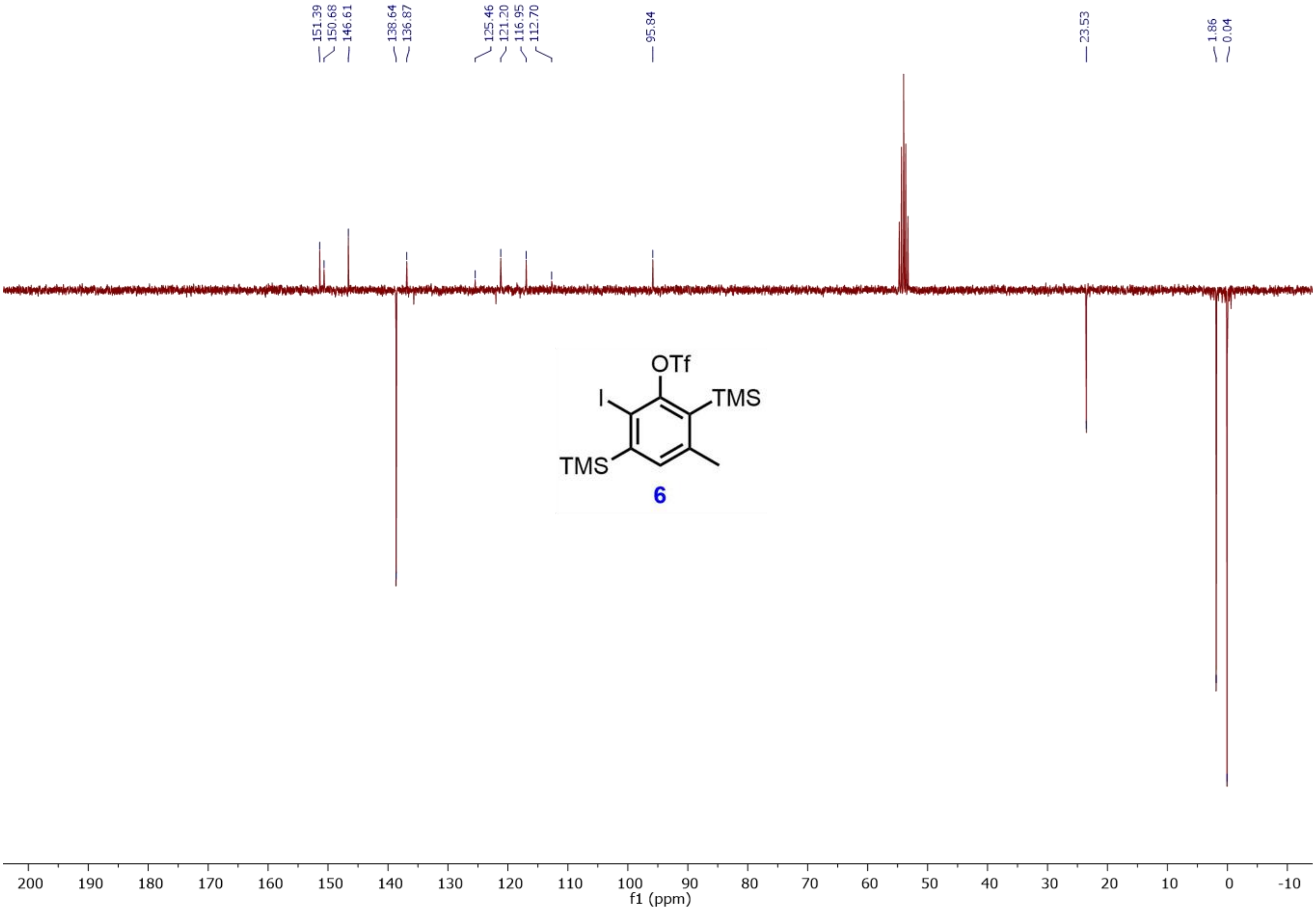
151.39
150.68
146.61
138.64
136.87
125.46
121.20
116.95
112.70
95.84
23.53
1.86
0.04
OTf
I
TMS
TMS
6
200
190
180
170
160
150
140
130
120
110
100
90
80
70
60
50
40
30
20
10
0
-10
f1 (ppm)

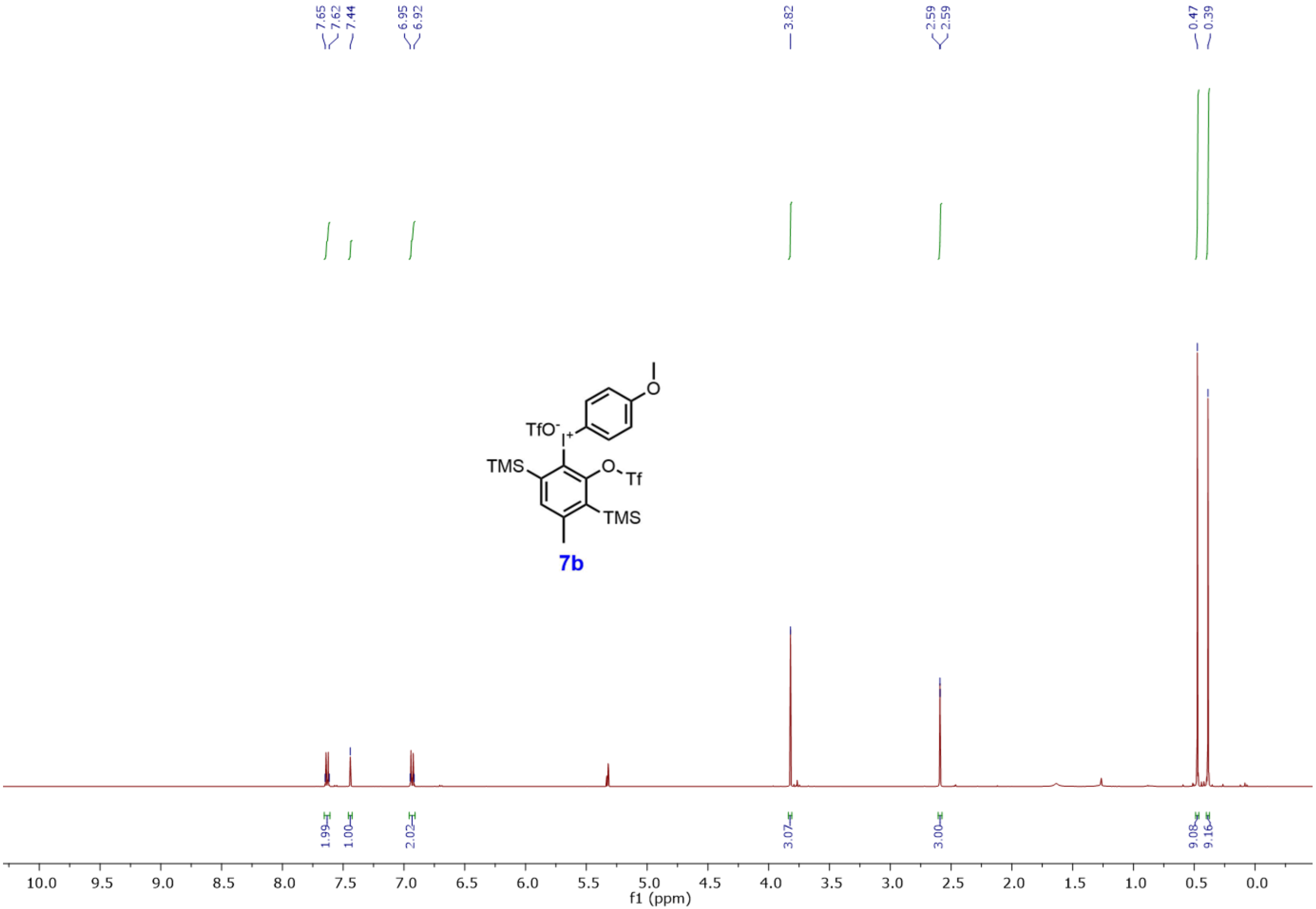
7.65
7.62
7.44
6.95
6.92
3.82
2.59
2.59
0.47
0.39
TfO-
I+
TMS
O
Tf
TMS
7b
1.99
1.00
2.02
3.07
3.00
9.08
9.16
10.0 9.5 9.0 8.5 8.0 7.5 7.0 6.5 6.0 5.5 5.0 4.5 4.0 3.5 3.0 2.5 2.0 1.5 1.0 0.5 0.0
f1 (ppm)

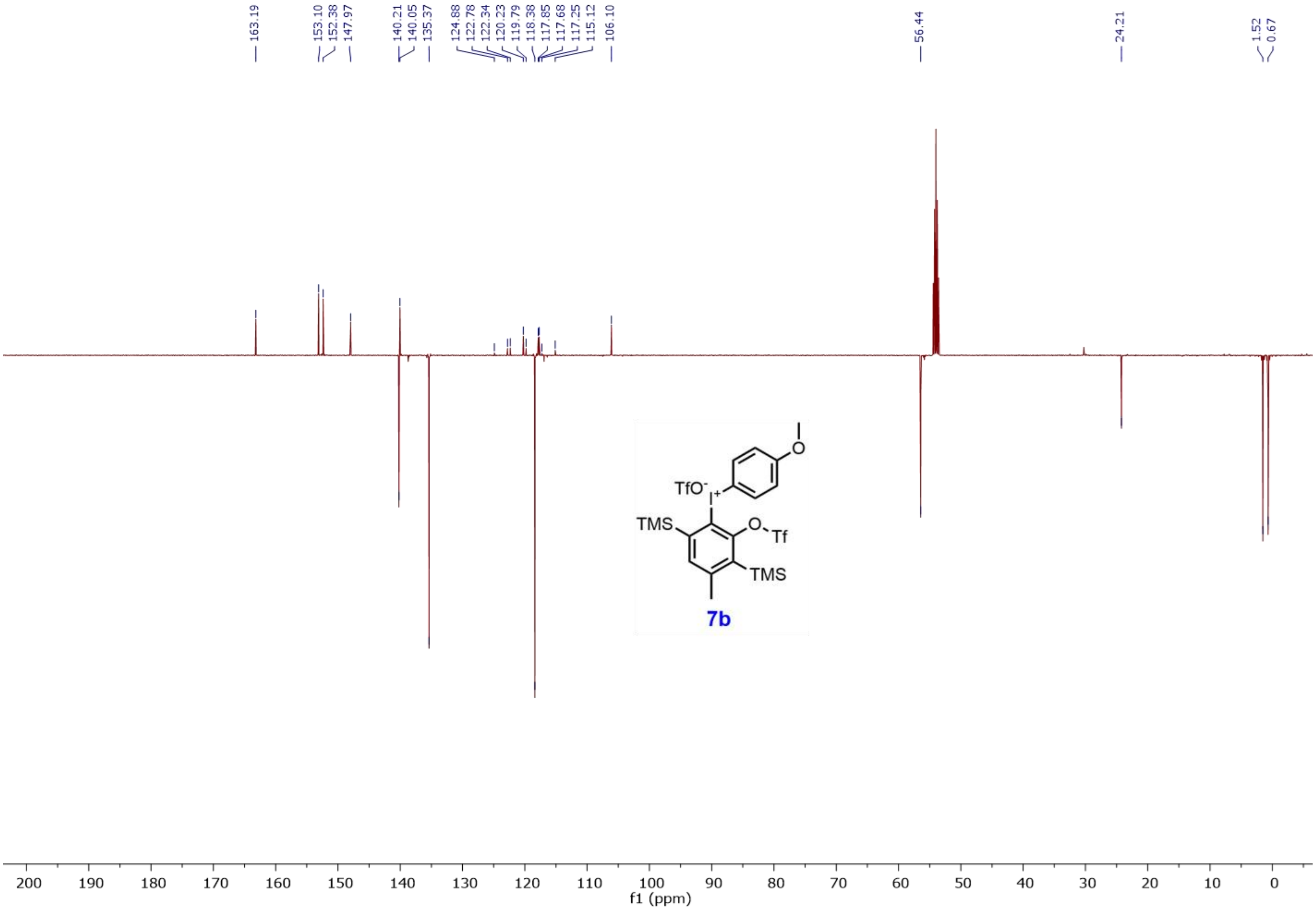

163.19
153.10
152.38
147.97
140.21
140.05
135.37
124.88
122.78
122.34
120.23
119.79
118.38
117.85
117.68
117.25
115.12
106.10
56.44
24.21
1.52
0.67
TfO⁻
I⁺
O
TMS
O
Tf
TMS
7b
200 190 180 170 160 150 140 130 120 110 100 90 80 70 60 50 40 30 20 10 0
f1 (ppm)

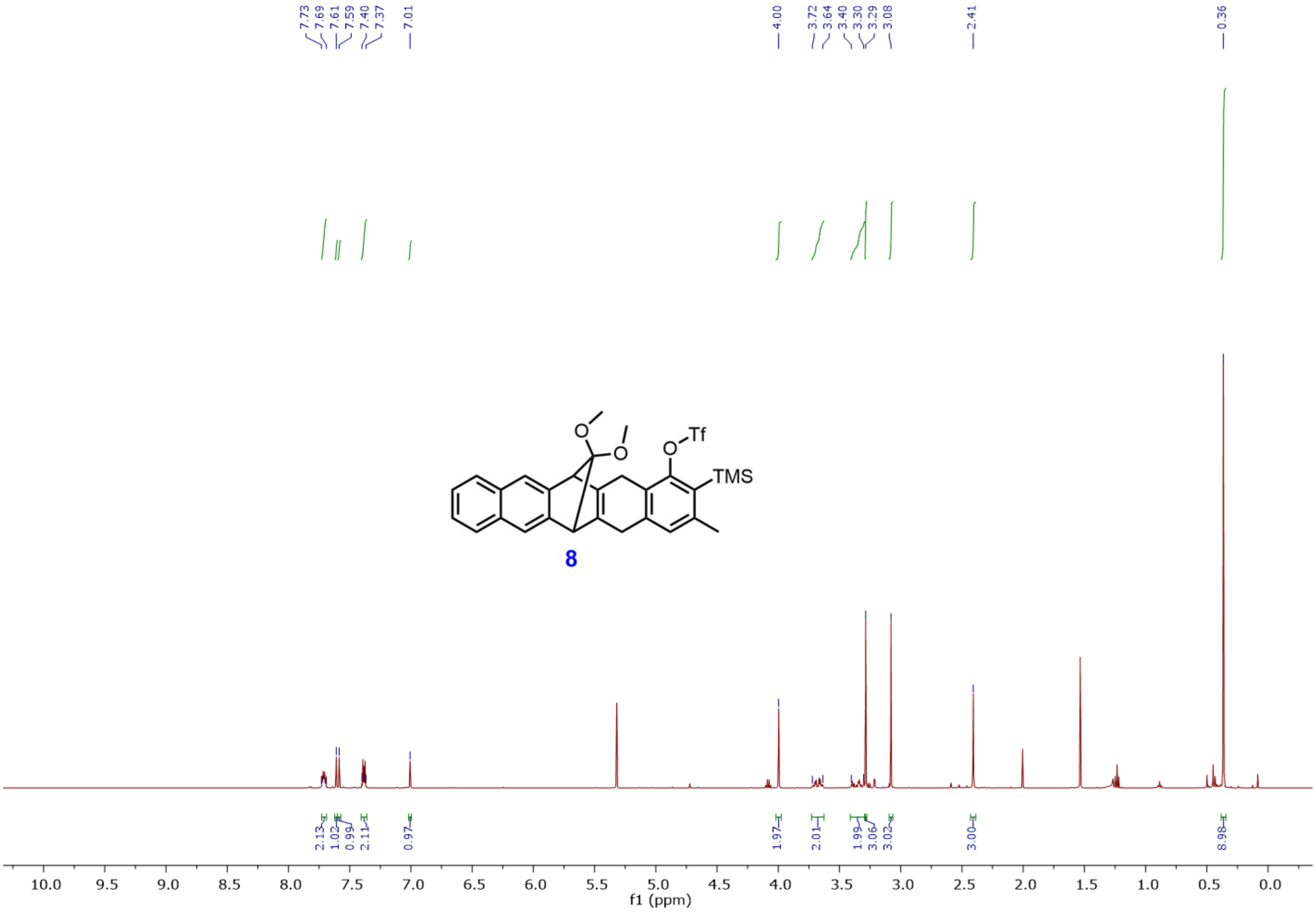
7.73
7.69
7.61
7.59
7.40
7.37
7.01
4.00
3.72
3.64
3.40
3.30
3.29
3.08
2.41
0.36
O
O
O
Tf
TMS
8
2.13
1.02
0.99
2.11
0.97
1.97
2.01
1.99
3.06
3.02
3.00
8.98
10.0
9.5
9.0
8.5
8.0
7.5
7.0
6.5
6.0
5.5
5.0
4.5
4.0
3.5
3.0
2.5
2.0
1.5
1.0
0.5
0.0
f1 (ppm)

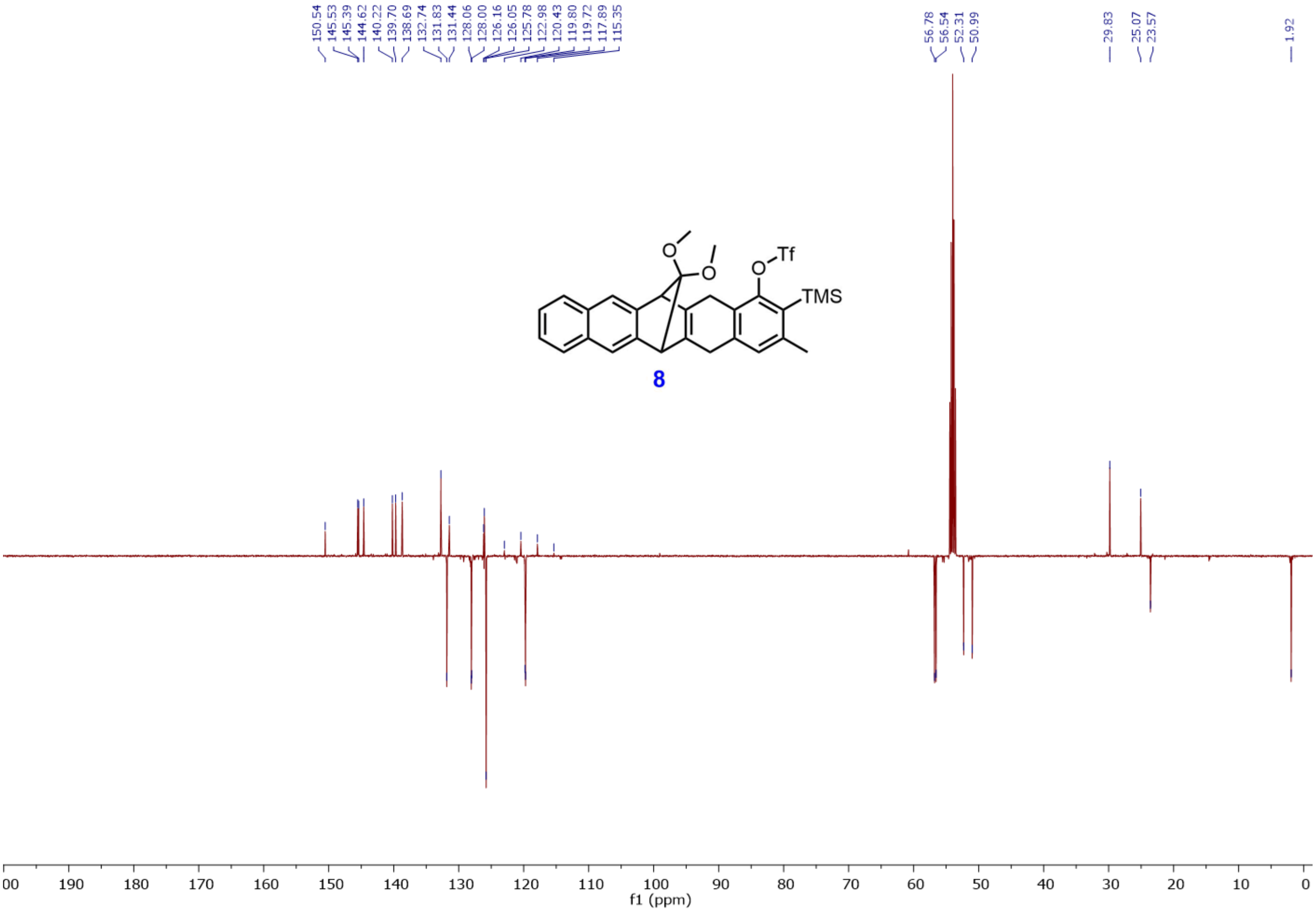
150.54
145.53
145.39
144.62
140.22
139.70
138.69
132.74
131.83
131.44
128.06
128.00
126.16
126.05
125.78
122.98
120.43
119.80
119.72
117.89
115.35
56.78
56.54
52.31
50.99
29.83
25.07
23.57
1.92
O
O
O
Tf
TMS
8
00
190
180
170
160
150
140
130
120
110
100
90
80
70
60
50
40
30
20
10
0
f1 (ppm)

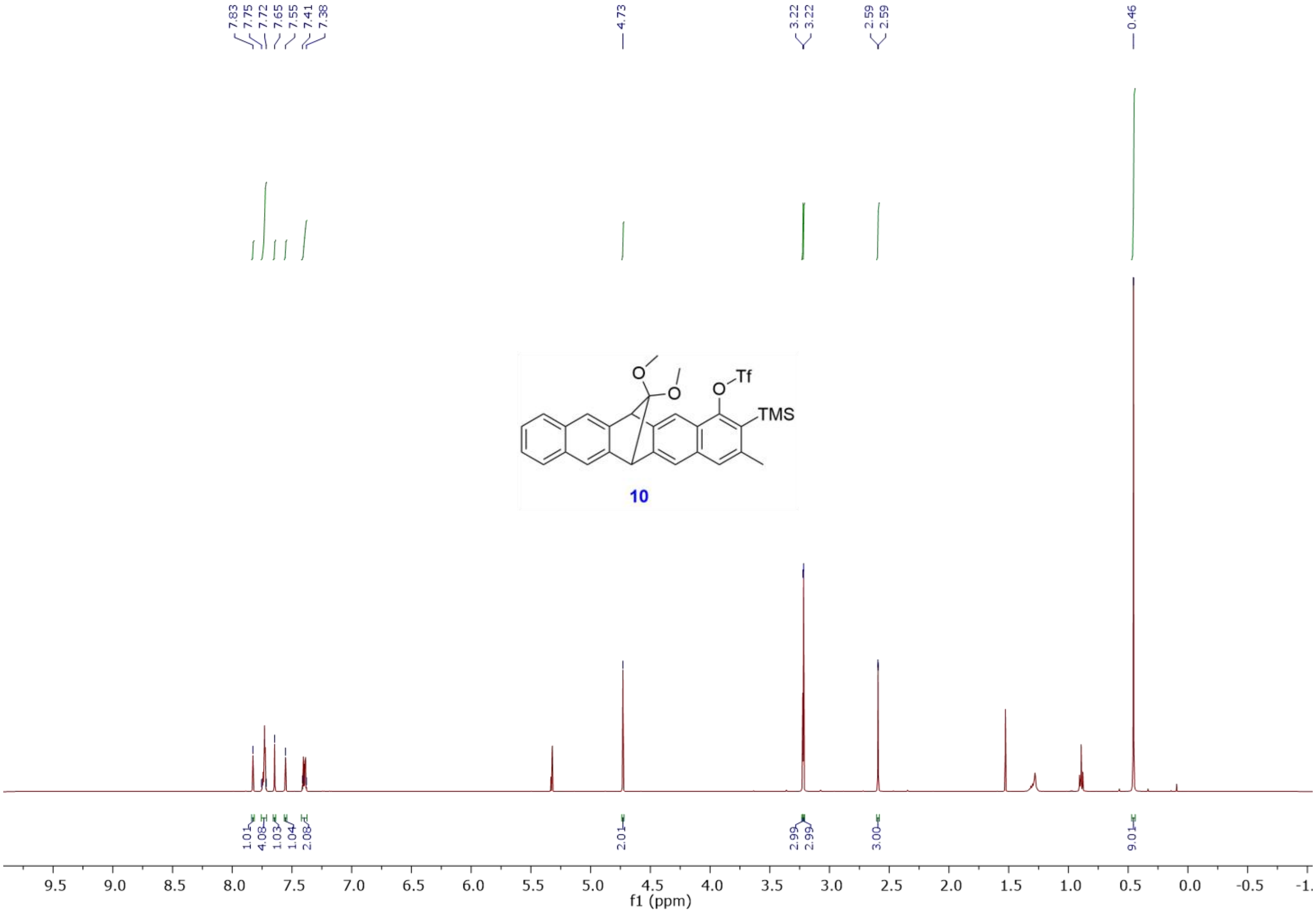
7.83
7.75
7.72
7.65
7.55
7.41
7.38
4.73
3.22
3.22
2.59
2.59
0.46
O
O
O
Tf
TMS
10
1.01
4.08
1.03
1.04
2.08
2.01
2.99
2.99
3.00
9.01
9.5
9.0
8.5
8.0
7.5
7.0
6.5
6.0
5.5
5.0
4.5
4.0
3.5
3.0
2.5
2.0
1.5
1.0
0.5
0.0
-0.5
-1.
f1 (ppm)

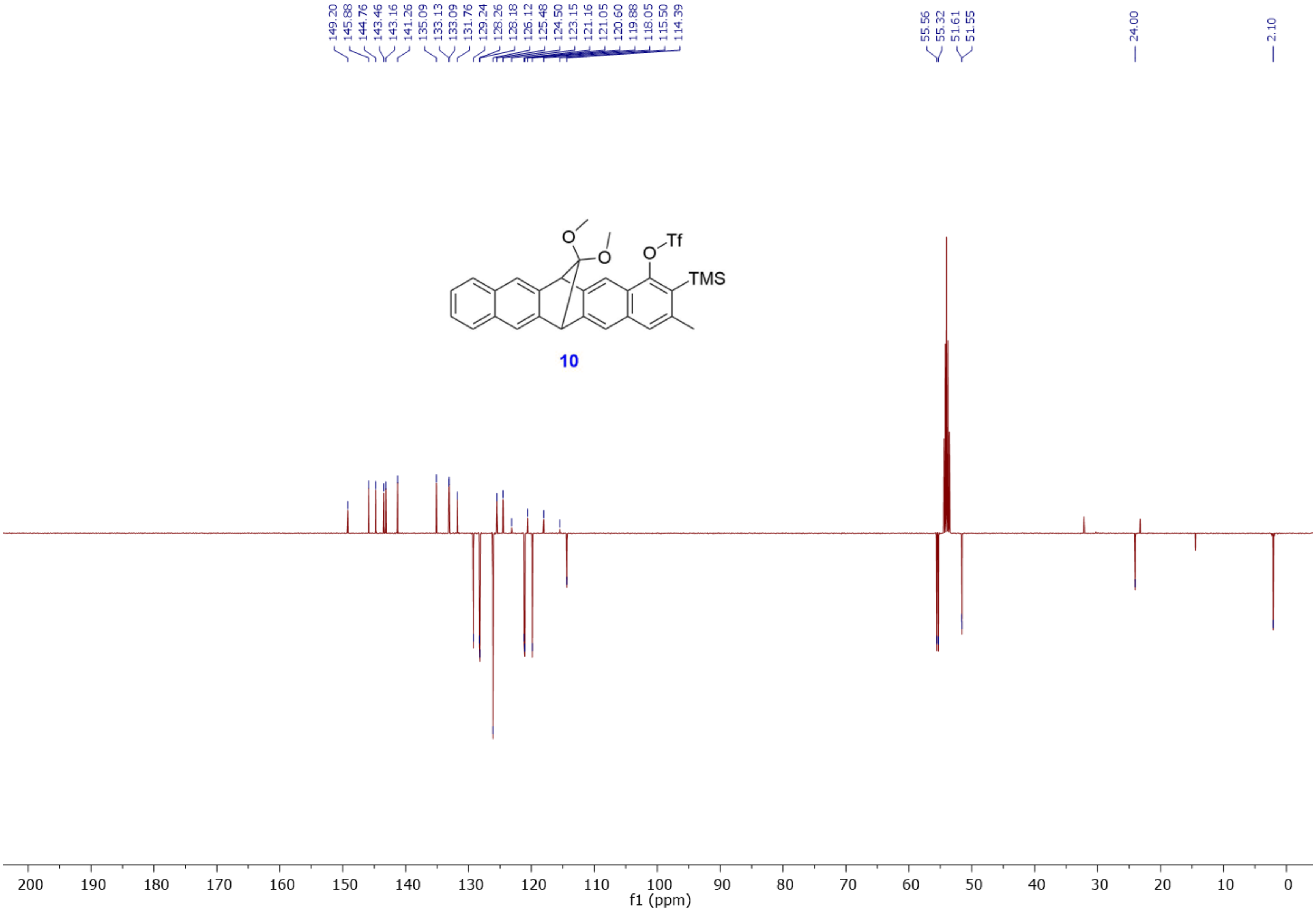

149.20
145.88
144.76
143.46
143.16
141.26
135.09
133.13
133.09
131.76
129.24
128.26
128.18
126.12
125.48
124.50
123.15
121.16
121.05
120.60
119.88
118.05
115.50
114.39
55.56
55.32
51.61
51.55
24.00
2.10
O
O
O
Tf
TMS
10
200
190
180
170
160
150
140
130
120
110
100
90
80
70
60
50
40
30
20
10
0
f1 (ppm)

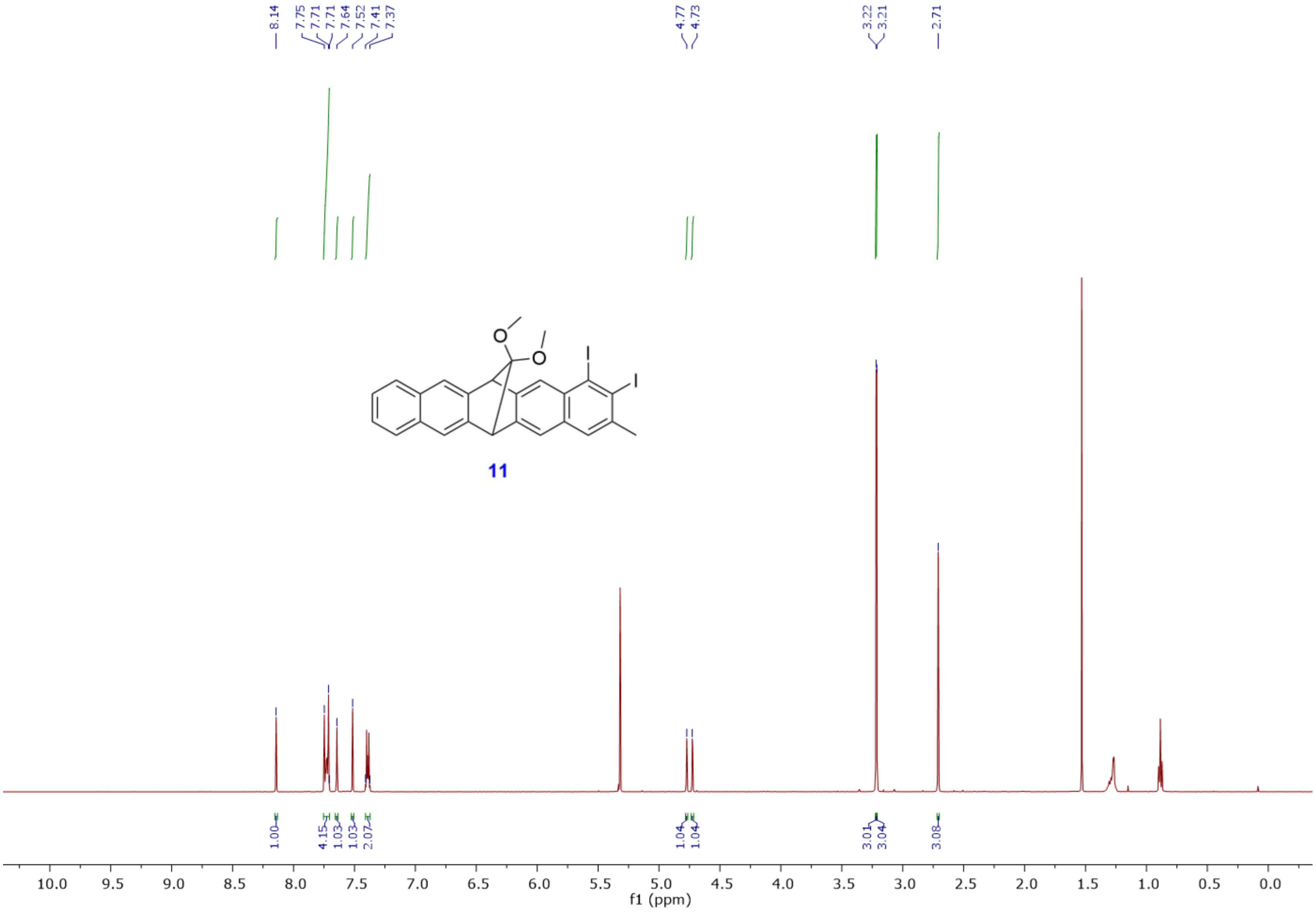
8.14
7.75
7.71
7.71
7.64
7.52
7.41
7.37
4.77
4.73
3.22
3.21
2.71
O
O
I
I
11
1.00
4.15
1.03
1.03
2.07
1.04
1.04
3.01
3.04
3.08
10.0 9.5 9.0 8.5 8.0 7.5 7.0 6.5 6.0 5.5 5.0 4.5 4.0 3.5 3.0 2.5 2.0 1.5 1.0 0.5 0.0
f1 (ppm)

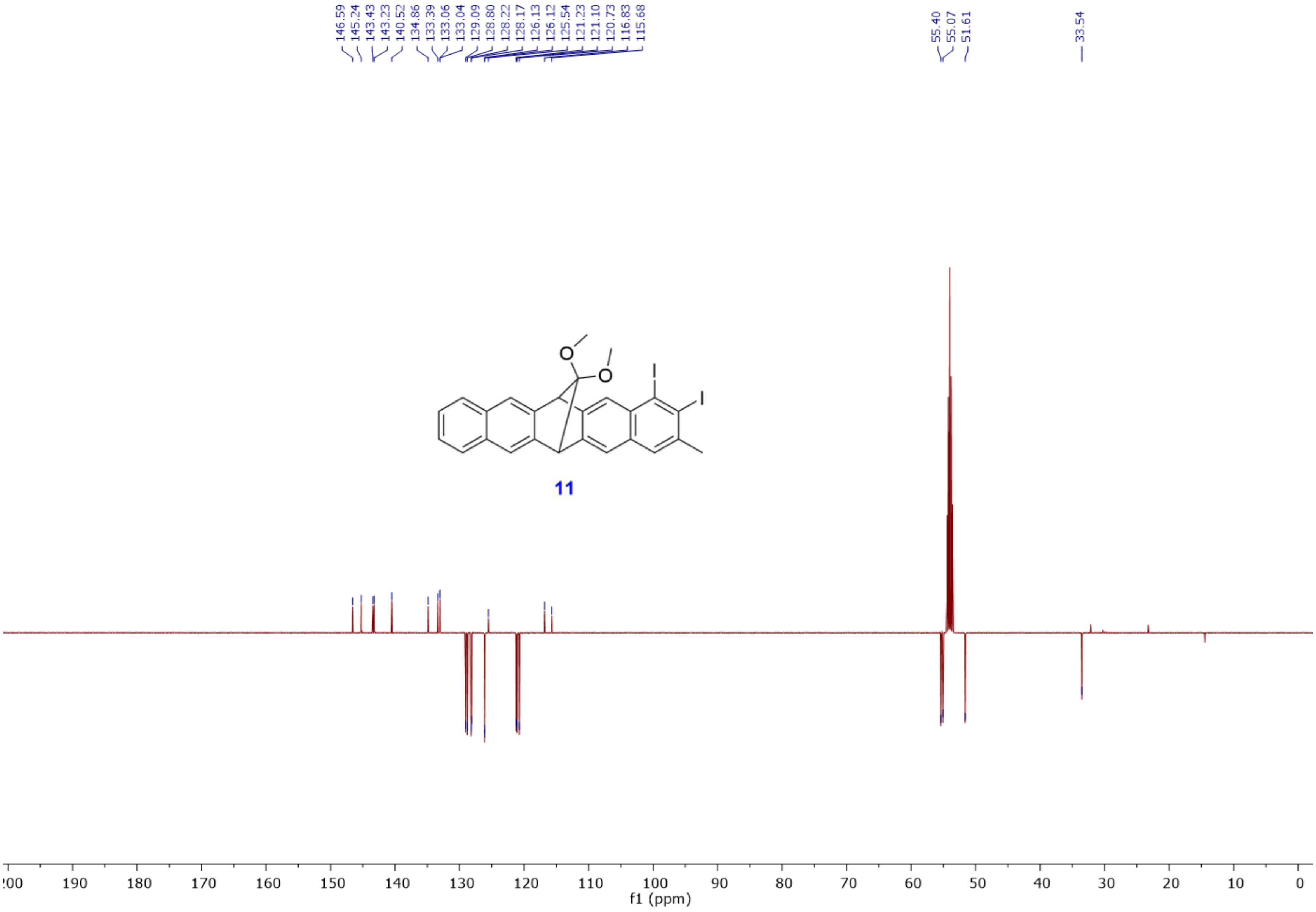
146.59
145.24
143.43
143.23
140.52
134.86
133.39
133.06
133.04
129.09
128.80
128.22
128.17
126.13
126.12
125.54
121.23
121.10
120.73
116.83
115.68
55.40
55.07
51.61
33.54
O
O
I
I
11
200
190
180
170
160
150
140
130
120
110
100
90
80
70
60
50
40
30
20
10
0
f1 (ppm)

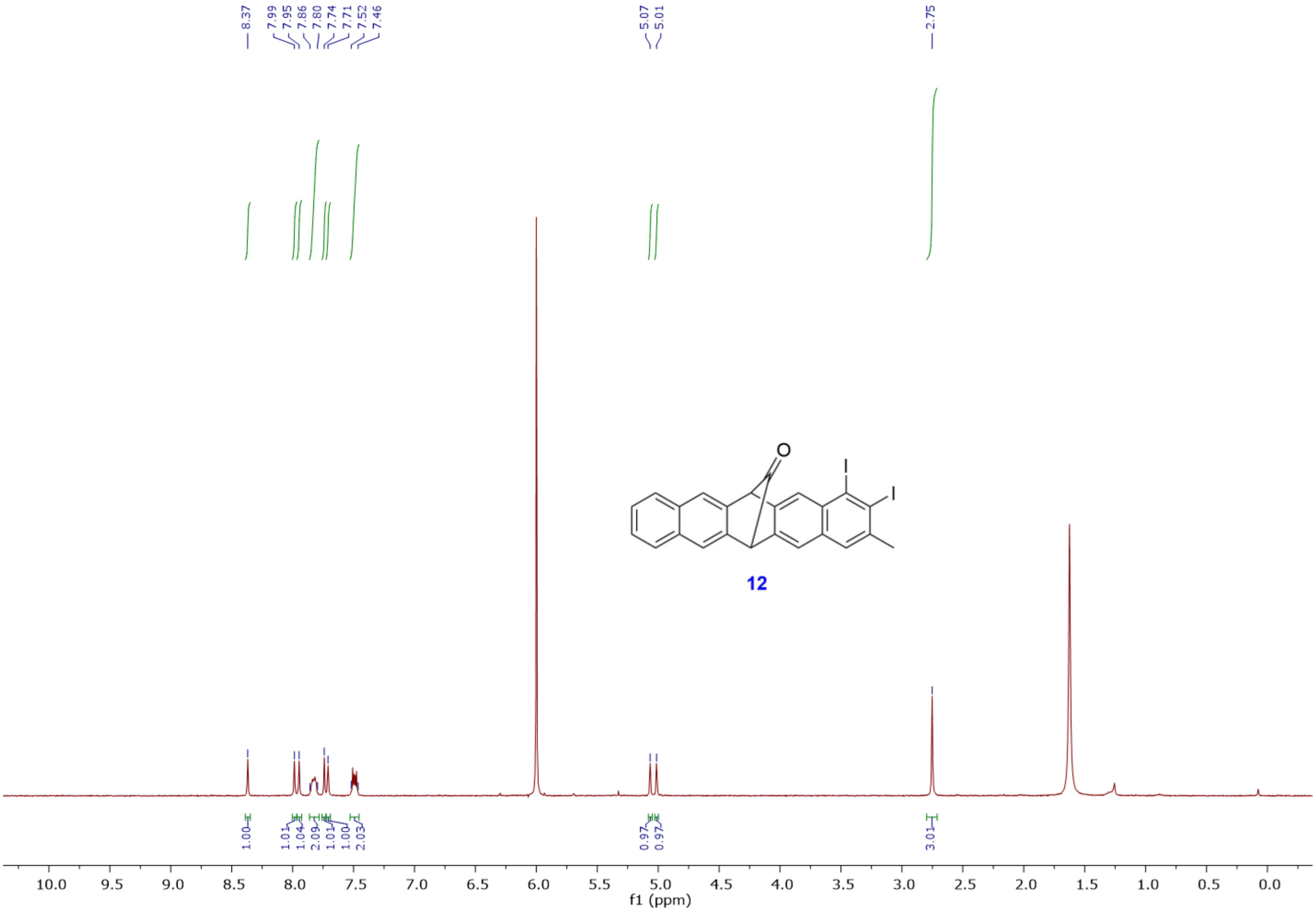

8.37
7.99
7.95
7.86
7.80
7.74
7.71
7.52
7.46
5.07
5.01
2.75
12
1.00
1.01
1.04
2.09
1.01
1.00
2.03
0.97
0.97
3.01
10.0 9.5 9.0 8.5 8.0 7.5 7.0 6.5 6.0 5.5 5.0 4.5 4.0 3.5 3.0 2.5 2.0 1.5 1.0 0.5 0.0
f1 (ppm)

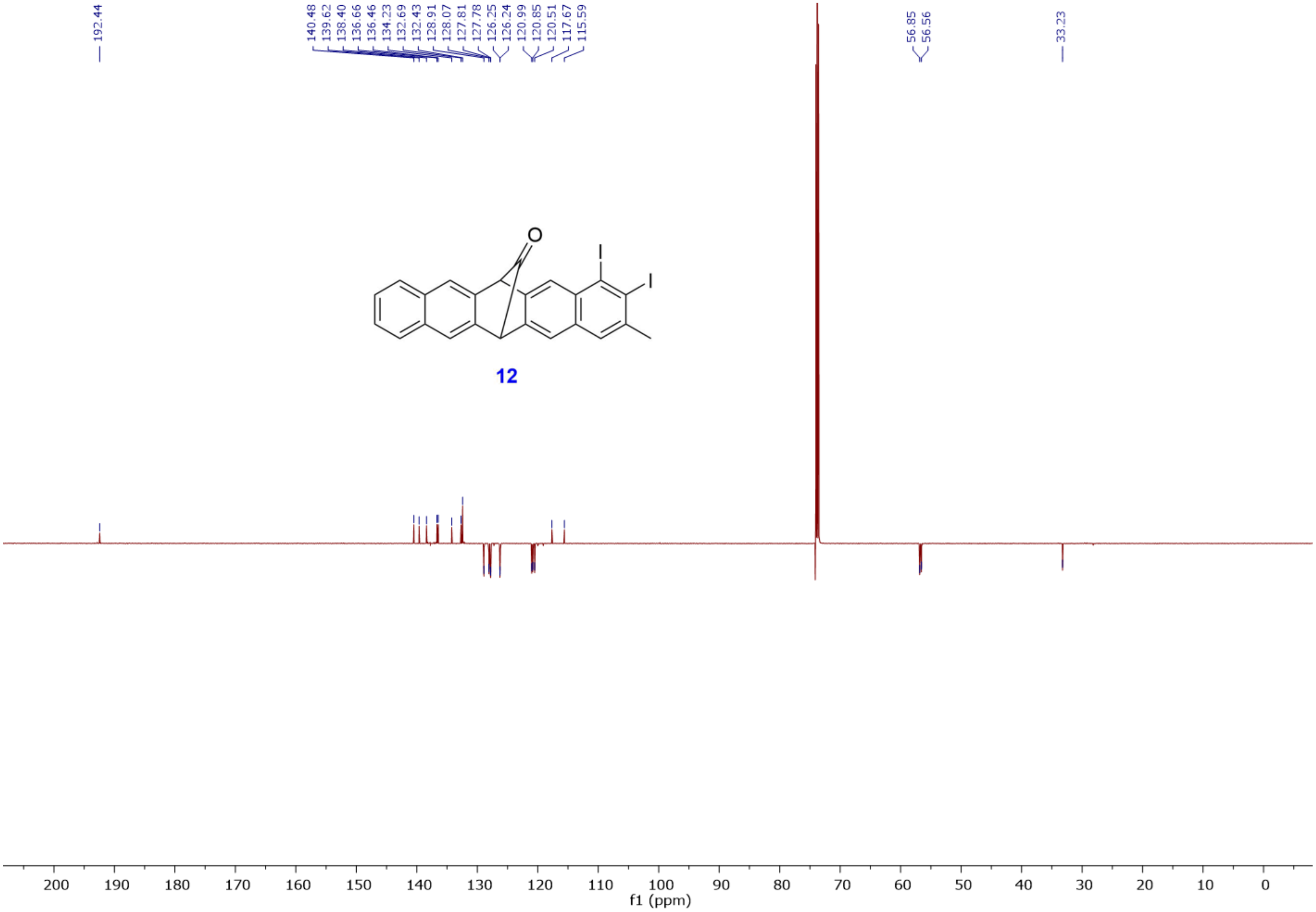
192.44
140.48
139.62
138.40
136.66
136.46
134.23
132.69
132.43
128.91
128.07
127.81
127.78
126.25
126.24
120.99
120.85
120.51
117.67
115.59
56.85
56.56
33.23
O
I
I
12
200
190
180
170
160
150
140
130
120
110
100
90
80
70
60
50
40
30
20
10
0
f1 (ppm)
